# Genome-wide analysis of light-regulated alternative splicing mediated by photoreceptors in *Physcomitrella patens*

**DOI:** 10.1186/gb-2014-15-1-r10

**Published:** 2014-01-07

**Authors:** Hshin-Ping Wu, Yi-shin Su, Hsiu-Chen Chen, Yu-Rong Chen, Chia-Chen Wu, Wen-Dar Lin, Shih-Long Tu

**Affiliations:** 1 Institute of Plant and Microbial Biology, Academia Sinica, Taipei 11529, Taiwan

## Abstract

**Background:**

Light is one of the most important factors regulating plant growth and development. Light-sensing photoreceptors tightly regulate gene expression to control photomorphogenic responses. Although many levels of gene expression are modulated by photoreceptors, regulation at the mRNA splicing step remains unclear.

**Results:**

We performed high-throughput mRNA sequencing to analyze light-responsive changes in alternative splicing in the moss *Physcomitrella patens*, and found that a large number of alternative splicing events were induced by light in the moss protonema. Light-responsive intron retention preferentially occurred in transcripts involved in photosynthesis and translation. Many of the alternatively spliced transcripts were expressed from genes with a function relating to splicing or light signaling, suggesting a potential impact on pre-mRNA splicing and photomorphogenic gene regulation in response to light. Moreover, most light-regulated intron retention was induced immediately upon light exposure, while motif analysis identified a repetitive GAA motif that may function as an exonic regulatory *cis* element in light-mediated alternative splicing. Further analysis in gene-disrupted mutants was consistent with a function for multiple red-light photoreceptors in the upstream regulation of light-responsive alternative splicing.

**Conclusions:**

Our results indicate that intensive alternative splicing occurs in non-vascular plants and that, during photomorphogenesis, light regulates alternative splicing with transcript selectivity. We further suggest that alternative splicing is rapidly fine-tuned by light to modulate gene expression and reorganize metabolic processes, and that pre-mRNA *cis* elements are involved in photoreceptor-mediated splicing regulation.

## Background

Alternative splicing (AS) is a widespread mechanism in eukaryotes that generates two or more mRNAs from the same precursor mRNA (pre-mRNA) by using different splice sites. The mRNA splicing process is executed by the spliceosome, which consists of RNAs and more than 180 protein subunits. Intron recognition by spliceosomes is directed by intron-defining *cis* elements, including consensus 5′ and 3′ splice sites, branchpoint, and polypyrimidine tract [1]. Many splice sites are bound by spliceosomes for constitutive splicing, but others are selectively recognized for AS. Selectivity of alternative splice sites is influenced by regulatory *cis* elements located in exonic and intronic regions, named splicing enhancers or repressors. These sequences are recognized by *trans*-acting splicing regulators such as serine/arginine-rich (SR) proteins and heterogeneous nuclear ribonucleoproteins (hnRNPs) [2,3]. Binding of these proteins to splicing enhancers or repressors can promote or inhibit the recruitment of splicing machinery to alternative splice sites. The expression of splicing regulators is differentially regulated in different cell types, tissues, developmental stages, and environmental conditions. The presence of regulatory *cis* elements on pre-mRNA, dynamic binding of *trans* factors, differential expression, and post-translational modification of splicing regulators collectively determine the AS patterns of expressed genes and eventually generate tremendous transcriptome and proteome complexity [3,4]. Indeed, recent genome-wide studies have shown that 42% to 61% of intron-containing genes in *Arabidopsis* and 33% to 48% of annotated genes in rice are alternatively spliced [5-7]. Many alternatively spliced transcripts contain premature termination codons (PTCs) that potentially leads to unproductive transcripts, truncated proteins or mRNA decay [8,9]. Therefore, AS also provides another level of gene regulation by modulating protein productivity, function, and transcript stability.

As sessile organisms, plants are constantly exposed to changes of environmental conditions. Light is one of the most crucial factors influencing plant growth and development. Fluctuation of light conditions affects the photosynthesis efficiency and rapidly stimulates developmental changes throughout the life cycle of plants. For example, when plant seedlings protrude from the soil (escape from etiolated state), developmental programs collectively called photomorphogenesis are rapidly turned on by light for vegetative growth. Plants have evolved sophisticated photoreceptor systems to regulate developmental processes of plant cells [10,11]. Plant photoreceptors include phytochromes, cryptochromes, phototropins, and ultraviolet (UV)-B photoreceptors. Phytochromes mainly perceive red and far-red light of the spectrum, whereas cryptochromes and phototropins perceive blue/UV-A light. Plant phytochromes can be further classified into two types, phytochrome B (phyB) and phytochrome A (phyA), according to their physiological responsiveness to red and far-red light, respectively [12]. Light perception by these photoreceptors triggers many biological processes, including gene regulation [11]. Global gene expression is rapidly altered by signal transduction or nuclear localization of light-activated photoreceptors in response to light changes.

Accumulating data suggest that light regulation can occur at different stages of gene expression to control the abundance of functional gene products. Chromatin modification has been found tightly regulated by light [13,14]. A variety of chromatin modifications on light-responsive gene loci have been identified. Such light-regulated dynamic alternations affect chromatin structure and play important roles in regulating transcriptional activity. At the molecular level, several light signaling components were proposed to function in chromatin regulation. A nuclear protein De-etiolated 1 (DET1) was shown to interact with UV-Damaged DNA Binding Protein 1 (DDB1) to regulate gene expression via chromatin binding [15]. In addition to chromatin regulation, light-regulated gene expression largely relies on the actions of light-signaling transcription factors (TFs), which have been extensively studied in the past few decades [16]. Although many TFs are involved in light signaling downstream of photoreceptors, the basic helix-loop-helix TF phytochrome interacting factors (PIFs), the basic zipper (bZIP) TF Long Hypocotyl 5 (HY5) and HY5 homolog (HYH) play a central role in integrating light signals to regulate light-responsive mRNA levels [17-19]. Light also regulates gene expression through translational control. Transcripts of photosynthetic genes are the target of light-mediated translational control [20-23]. More recently, a global survey of transcripts under translational regulation during photomorphogenesis in *Arabidopsis* has been reported [24]. Furthermore, light post-translationally affects abundance of key regulators through ubiquitin-dependent protein degradation. The CONSTITUTIVE PHOTOMORPHOGENIC 1 (COP1), an E3 ubiquitin ligase, target key components in light signaling for proteolysis via 26S proteasome pathway [25]. The COP9 signalsome (CSN), a protein complex with eight subunits in ubiquitin-proteasome pathway, is also involved in proteolysis of photomorphogenesis regulators in plants [26]. Although light regulation is found in almost every stage of gene expression, information on light regulation in the mRNA splicing step is relatively lacking.

In plants, many gene transcripts undergo AS in response to abiotic and biotic stresses [27]. Several reports have indicated that the abundance of alternatively spliced transcripts is altered by changing light conditions [28-30]. In addition, alternatively spliced transcripts of light signaling genes can function differently in light responses. Overexpression of an alternative isoforms of PIF6 in *Arabidopsis* was shown to elevate the efficiency of seed germination [31]. An alternatively-spliced isoform of COP1 also negatively regulate COP1 function in photomorphogenesis [32]. These results reveal that AS of light signaling genes can be important in regulating light responses. Most notably, a recent report identified an *Arabidopsis* mutant, *reduced red-light responses in cry1cry2 background 1* (*rrc1*), with impaired phyB-dependent photomorphogenic responses [33]. *RRC1* encodes a potential splicing factor with a C-terminal arginine/serine-rich (RS) domain that is important for phyB signaling. Further analysis in this report showed that AS patterns of SR protein gene transcripts are altered by red light and this effect is reduced in *phyB* and *rrc1* mutants. These data suggest that photomorphogenic responses in plants can be controlled by gene regulation at the mRNA splicing step.

Although these reports indicate that AS of some genes can be affected by light in plants, genome-wide analysis of AS in light responses is still not available. In this study, we used high-throughput mRNA sequencing (RNA-seq) to detect transcriptome changes during light exposure for 1 or 4 h of red or blue light. We examined the responsiveness of AS at red and blue light wavelengths to investigate the possible involvement of red and blue light-dependent mechanisms for splicing regulation. A simple model system, the moss *Physcomitrella patens*, was adopted for this approach. In general we found around 50% of moss genes undergoing AS in the simple-cell-type protonema. Intron retention (IR) is the most prevalent form of AS in *Physcomitrella*. Light-regulated AS events were identified with a statistic method and experimentally validated. Analysis of the top 1,000 light-regulated IR events showed that IR of most genes were rapidly induced within 1 h, especially for genes functioning in chloroplast and translation. Furthermore, AS of many splicing-related and light signaling gene transcripts were also regulated by light. Comparison of IR pattern for wild type and a phytochrome-deficient mutant reveal the involvement of photoreceptors in immediate regulation of AS. Further analysis in gene-disrupted phytochrome mutants indicates that the phyB type of moss phytochromes is responsible for splicing regulation under red light. Altogether, our results suggest that light-regulated AS is important in shaping transcriptome for light responses. The splicing machinery is rapidly responsive to light. Photoreceptors are activated by specific wavelength of light to regulate AS, which results in the production of functional proteins for gene regulation and cellular processes during photomorphogenesis.

## Results

### RNA-seq identified a substantial number of reads supporting AS in *Physcomitrella*

In a previous study, we reported transcriptome changes in *Physcomitrella* in response to 1-h red light [34]. To further understand the light responsiveness in non-vascular plants, we expanded our approach with longer red-light exposure and with blue-light wavelength. Two-week-old *Physcomitrella* protonema were grown in the dark for 3 days (dark-grown control, D), then exposed to constant red (Rc) or blue light (Bc) for 1 (R1 and B1) and 4 h (R4 and B4). The expression of light-responsive marker genes was first checked by RT-PCR (see Additional file 1: Figure S1). For RNA-seq, total RNA pooled from three biological replicates for each sample were subjected to cDNA library preparation (see Additional file 1: Figure S2). Sequencing involved the Illumina Hiseq 2000 platform with the paired-end (100-nt read length) method. After data trimming and filtering, nearly 195.5 million reads were generated (see Additional file 1: Table S1). Sequence reads were mapped to the *Physcomitrella* genome annotation V1.6 [35]. Approximately 138.4 million reads (70.79% of total reads) were perfectly aligned to the reference genome, and 91% matched annotated gene regions. Reads mapped to the annotated gene regions were counted to calculate the reads per kilobase of exon model per million mapped reads (RPKM) for estimating gene expression [36].

Among the mapped reads, we found nearly 4.3 and 4.9 million reads located in the annotated intronic regions and across the exon-intron junctions, respectively. These reads were potentially generated from alternatively spliced transcripts in each sample. To further dissect these reads into different AS categories, we used the RACKJ package to isolate the reads supporting different AS events and statistically measure the light sensitivity of the event [37,38]. Reads aligned to AS sites for each sample were counted. Data from red- (R1 and R4) and blue-light (B1 and B4) samples were compared with the dark-grown control (D). To eliminate false-positive events, we filtered AS events originally detected by the following three criteria stepwise. In initially detected events from red- and blue-light samples, their corresponding genes with <2 reads mapped to exon regions were first removed; the remained events with ≥2 reads aligned to splice junction(s) of AS sites were then retained; the events with at least 2 reads supported were then included in the final list. Chi-square test was used to compare read counts supporting and not supporting the AS event in each sample. For example, to calculate the significance of a red-light-regulated IR event, the number of intronic reads was compared with read numbers for corresponding gene exons (representing total transcripts) in D, R1, and R4 samples. Similarly, to calculate the significance of an exon skipping (ES) event, exon-skipped read counts supporting this event were compared with read counts aligned to the skipped exon. For the alternative donor site/alternative acceptor site (AltD/AltA) events, AltD/AltA read counts were then compared to constitutive donor and acceptor read counts. With this statistical method, the light sensitivity of an event could be quantified by normalization to the expression level of a corresponding gene or flanking exons.

### Frequencies of AS events in moss protonema are comparable to that in higher plants

**Figure 1:**
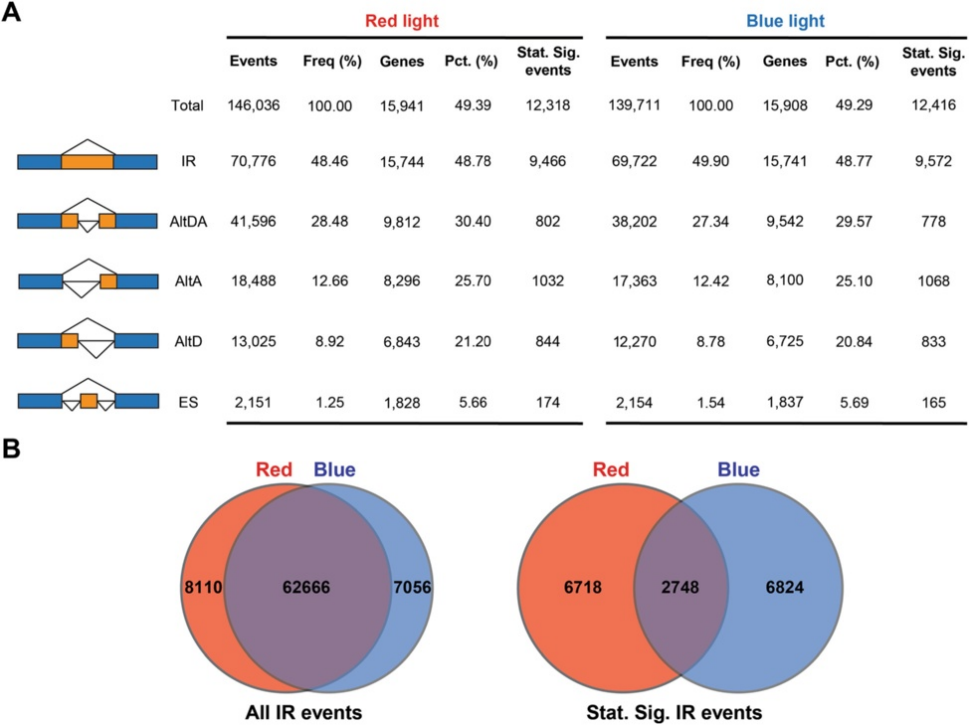
**Alternative splicing (AS) events found in *Physcomitrella*. (A)** Total AS events and top five most common types of AS. The intron-exon structure of the AS events and its description is illustrated on the left. In each table, the first column represents the initial number of events found in combined samples, then the frequency of the event among all AS events, the number and percentage of moss genes (annotated: 32,272) showing the event, and the number of statistically significant events. AS events were statistically significant at *P* <0.001. AltA, alternative acceptor site; AltD, alternative donor site; AltDA, alternative donor/acceptor site; ES, exon skipping; IR, Intron retention. **(B)** Venn diagrams of IR events in red- and blue-light samples. The diagrams compare all IR events (left) and statistically significant IR events (right) found in red- and blue-light samples.

Although our RNA-seq data were mainly generated from light-treated samples, we believe the sequencing depth can reflect frequencies of AS events occurring in *Physcomitrella*. We detected totally 146,036 and 139,711 AS events in red- and blue-light samples, respectively (Figure 1A). Nearly 50% of *Physcomitrella* genes were alternatively spliced in the protonema stage. Five major types of AS events were found: IR, ES, AltD, AltA, and AltD/AltA (AltDA) (Figure 1A and see Additional files 2 and 3). The number of events identified from red- and blue-light samples was surprisingly close. We compared all IR events identified in red- (70,776) and blue- (69,722) light samples. Nearly 90% of IR events in both samples overlapped (Figure 1B). The data not only reveal the consistency of RNA-seq experiments, but also suggest that our AS survey covered the majority of AS events exist in *Physcomitrella*. However, when we compared the IR events with statistical significance in response to red (9,466) and blue (9,572) light, only <30% of events were found responsive to both conditions. This result indicates that the events sensitive to light treatments are differentially regulated. A large portion of IR events is specifically responsive only to red or blue light.

In *Physcomitrella*, IR is the most common event, with a frequency of nearly 50% (Figure 1A). AltD and AltA are less abundant than IR, with higher usage of AltA than AltD. In addition, AltDA is more active than AltD and AltA. ES events identified in each light sample are only present in about 6% of moss genes. We conclude that the general pattern of AS is conserved among land plants. Complex and diverse AS events can be found in the simple-cell-type protonema of the ancient plant species.

### Light-responsive IR occurs on specific gene transcripts

Similar to higher plants, transcription of moss genes are rapidly regulated in response to light changes [34]. AS may also play an important role in modulating the abundance of productive transcripts for light responses. Since IR is the most prevalent event responsive to the light, we first investigated the biological functions of light-responsive IR events. Because more than 9,000 IR events were statistically significant, we selected the top 1,000 events for subsequent analysis. Corresponding genes of the top 1,000 IR events responsive to Rc and Bc were subjected to functional enrichment analysis and classified according to Gene Ontology (GO) terms. Over-represented GO terms among functionally annotated genes are in Table 1. In both red- and blue-light data, translation-related terms were largely represented. Of these, genes encoding ribosomal proteins were highly enriched. In contrast, photosynthesis-related terms were enriched significantly in the top 1,000 Bc-responsive IR events, which implies that blue light immediately regulates IR of transcripts necessary for photosynthetic growth in the light. Our results strongly suggest that AS of transcripts involved in specific functions can be rapidly and differentially regulated by light.

**Table 1:** Over–represented Gene Ontology (GO) terms of top 1,000 intron retention (IR) genes regulated by red and blue light

** Red light**	
**GO term**	***P *** **value**
MF: structural constituent of ribosome	3.95E–29
CC: ribosome	9.50E–25
BP: translation	1.22E–22
CC: integral to membrane	1.19E–06
BP: cell redox homeostasis	1.96E–06
CC: mitochondrion	8.95E–05
MF: glycerone kinase activity	9.31E–05
A total of 1,000 genes were submitted to the GOBU functional enrichment tool, which resulted in 689 unique genes with annotations. Terms are ranked by *P* value of over–representation and are included in list if *P* <1E–04. BP: Biological process; CC: Cellular component; MF: Molecular function.	
** Blue light**	
**GO term**	***P *** **value**
CC: chloroplast	4.67E–33
MF: structural constituent of ribosome	1.66E–29
CC: ribosome	5.14E–27
BP: translation	1.63E–20
BP: reductive pentose–phosphate cycle	1.47E–16
CC: photosystem II	2.36E–10
BP: photosynthesis, light harvesting	6.62E–10
CC: photosystem I	8.56E–10
CC: stromule	2.32E–09
MF: chlorophyll binding	2.53E–08
CC: thylakoid	6.47E–08
BP: photorespiration	1.00E–07
CC: chloroplast envelope	1.12E–07
BP: photosynthesis	3.92E–07
CC: oxygen evolving complex	5.16E–07
CC: cytosolic ribosome	3.22E–06
MF: rRNA binding	1.68E–05
BP: cell redox homeostasis	2.21E–05
BP: translational elongation	2.26E–05
MF: ribulose–bisphosphate carboxylase activity	2.86E–05
BP: nitrate assimilation	5.10E–05
MF: glycerone kinase activity	7.63E–05

A total of 1,000 genes were submitted to the GOBU functional enrichment tool, which resulted in 733 unique genes with annotations. Terms are ranked by *P* value of over–representation and are included in list if *P* <1E–04.

BP: Biological process; CC: Cellular component; MF: Molecular function.

**Figure 2:**
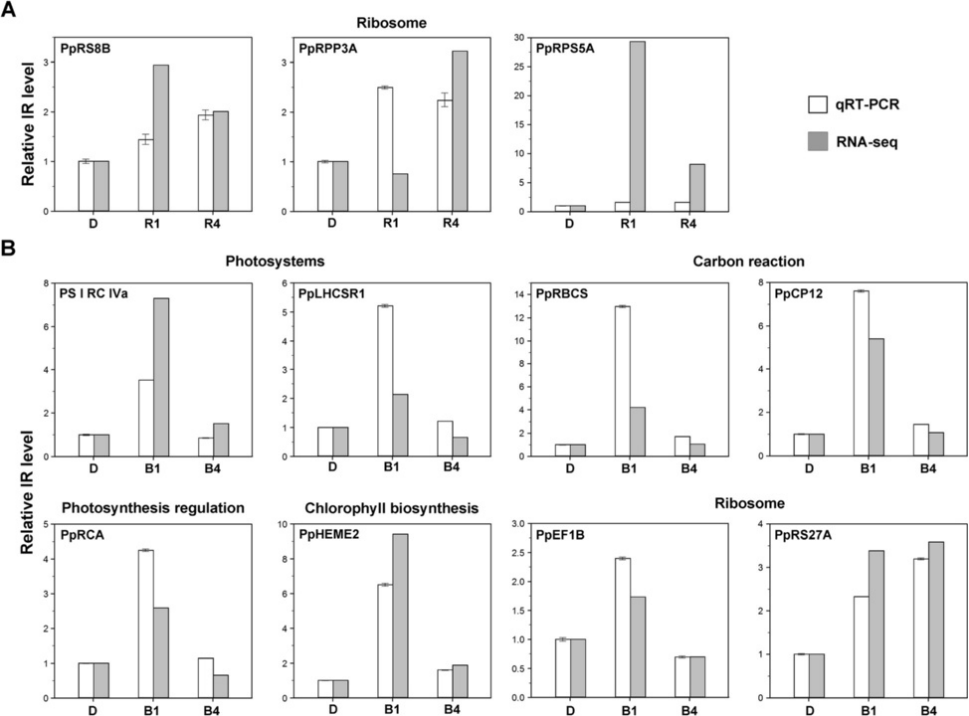
**Validation of light-regulated intron retention (IR) events.** Pooled RNA from dark-grown (D), 1-h light-treated (R1 and B1), 4-h ligh-treated (R4 and B4) samples were tested in triplicate for qRT-PCR. Primer sets designed for amplifying IR regions, total transcripts of the corresponding genes, and *PpACT2* were used (see Additional file 7). *PpACT2* was first used as an internal control for normalization of each qRT-PCR reaction. IR level were further normalized for total transcripts, and then compared with data from the dark-grown control to generate a relative IR level. IR events were selected from red- **(A)** and blue- **(B)** light samples. To determine the IR level from RNA-seq data, the level of IR isoforms was computed for the intron reads per kilobase of retained intron per million mapped reads (IPKMs). For comparison, IPKMs were normalized with RPKMs of corresponding genes and calculated for relative IR level compared to the dark-grown control. Corresponding gene products of selected IR events and representing processes are shown in each graph and above, respectively.

The IR level of representative events for these GO terms was further validated by quantitative RT-PCR (qRT-PCR). We designed primer pairs to specifically detect the IR level and total transcripts of the corresponding gene. The IR level was normalized for total transcripts and then compared with data from the dark-grown control to generate the relative IR level. To calculate the IR level from RNA-seq data, we computed the intron reads per kilobase of retained intron per million mapped reads (IPKM) for each event. IPKM values were then normalized to RPKM values of corresponding genes and calculated for relative IR level compared to the dark-grown control. Indeed, IR levels for the selected genes were differentially regulated under both light conditions (Figure 2A and B). Patterns of light responsiveness were mostly comparable between qRT-PCR and RNA-seq data. This result supports the validity of our statistical method for identifying light-sensitive AS events.

### IR is rapidly induced by light in specific subset of chloroplast and ribosomal protein genes

**Figure 3:**
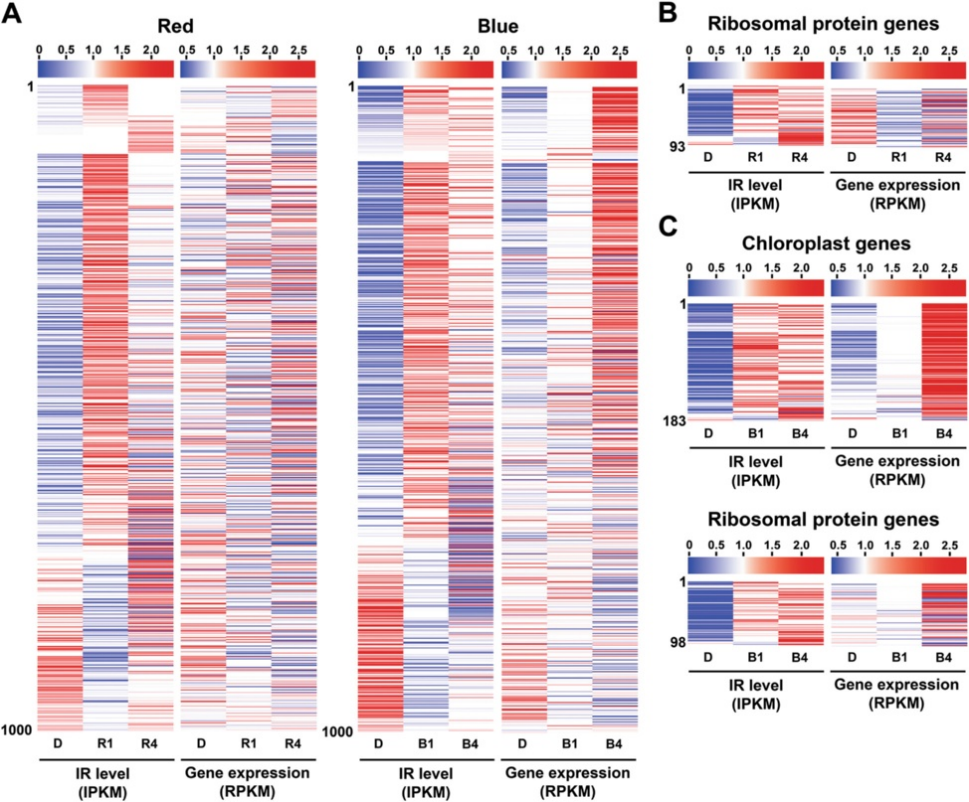
**IR is rapidly induced by light. (A)** Expression profiles of top 1,000 red (left) and blue (right) light-regulated IR isoforms and corresponding genes. IR level (IR) and corresponding gene expression level (Gene expression) for the top 1,000 IR events were normalized by mean across samples, respectively. Heat maps of these events in dark-grown (D), 1-h light-treated (R1 and B1), and 4-h light-treated (R4 and B4) samples are shown. Events were arranged according to the fold-change of IR level comparing 1-h light-treated to dark-grown samples. The scale of expression is shown on the top of each figure. Heat maps of expression levels of IR isoforms and corresponding genes encoding ribosomal proteins in red-light sample **(B)**, and chloroplast proteins and ribosomal proteins in blue-light sample **(C)** are shown.

Data from qRT-PCR analysis for the top 1,000 light-responsive IR events revealed an obvious light-sensitive pattern that most of IR events we validated showed immediate light induction at 1 h (Figure 2). To examine whether the pattern is common among light-responsive IR events, we compared IR levels (IPKMs) and corresponding gene expression levels (RPKMs) for the top 1,000 IR events. Similar to that observed by qRT-PCR data, more than 70% of the top 1,000 IR events were simultaneously upregulated within 1 h under both red and blue light, but their corresponding gene expression levels were overall unchanged (Figure 3A). This result demonstrates that light-activated IR is not associated with transcriptional activity. IR was induced immediately after exposure to light and became moderate in the later stage (4 h under light), which suggests that splicing of these introns was rapidly inhibited by light. Rapid IR induction was also found for chloroplast and ribosomal protein genes (Figure 3B and C), which further reveals that splicing inhibition occurred in transcripts for specific biological processes.

IR usually produces PTC and generates unproductive transcripts or truncated proteins. To further confirm that retained introns in IR transcripts generated PTCs, we computed the proportion of PTC-occurring IR transcripts. We found most of IR transcripts had potential PTCs in the retained intron or downstream regions (see Additional file 1: Table S2). These results suggest that light repress intron splicing for certain gene transcripts may generate unproductive species to temporally attenuate specific biological responses after light exposure.

### IR of splicing factor gene transcripts were regulated by light

**Figure 4:**
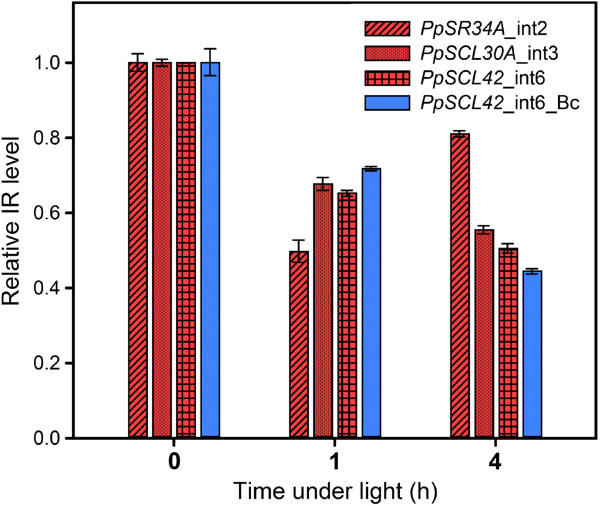
**IR of SR protein genes found in the top 1,000 light-regulated IR events.** Quantitative RT-PCR analysis of IR level in samples grown under the dark (0-h light), 1-h, and 4-h of red (red-colored bars) and blue (blue-colored bar) light are shown. IR level of SR protein genes were tested in triplicate with primer sets designed for IR isoforms, total transcripts of the corresponding genes, and *PpACT2* (see Additional file 7). Intron number (intNo) of the gene counted from 5′ of the genome is labeled after the gene name. Bc, constant blue light.

Plant genomes contain a large amount of splicing-related genes encoding core spliceosome components, splicing factors, and regulators [39]. Most of them are conserved among plants and metazoans. Many transcripts for these splicing-related proteins especially splicing regulators also show extensive AS patterns that are altered by environmental stresses [39,40]. Although splicing-related genes were not highly enriched in our data, those showing light-regulated AS events potentially impact on splicing regulation for light responses. We surveyed the lists of genes with IR events responsive to red and blue light and found more than 70 splicing-related genes in red- and blue-light data, respectively. These genes can be categorized into several groups encoding SR proteins, KH-domain proteins, polypyrimidine tract binding proteins (PTBPs), DEAD box helicases, spliceosome components, and other splicing factors (see Additional files 4 and 5). We selected SR protein genes in the top 1,000 events of both red- and blue-light data for validation by qRT-PCR (Additional file 1: Figure S5). Interestingly, although majority of the top 1,000 IR events were rapidly induced by light, IR for all of these genes showed the pattern of light repression (Figure 4). These results suggests that light enhance splicing activity for these genes to generate productive transcripts, which allow to synthesize more functional SR proteins for subsequent splicing regulation under the light.

### Light signaling genes were alternatively spliced for photomorphogenic control

In *Arabidopsis*, alternatively spliced isoforms of light signaling genes have been found involved in plant development [31,32]. However, whether AS of light signaling gene transcripts can be modulated is unknown. Light signaling genes in our AS data may play important roles in photomorphogenesis. After surveying potential light signaling genes in moss genome and those showing light-regulated AS in our data, we found 36 of them alternatively spliced under red or blue light (Table 2 and see Additional file 6). These genes cover almost all the steps of photomorphogenic gene regulation from chromatin remodeling to post-translational protein degradation. To our surprise, almost all members of several light signaling gene families including *HY5/HYH*, *PIFs*, *COP1*, *CSNs*, *DET1*, and *DDB1* underwent light-regulated AS. AS regulation for these genes potentially modulate the function or abundance of light signaling factors, and could impact on both red- and blue-light signaling pathways.

**Table 2:** **Light signaling genes in *Physcomitrella *** **showed light-regulated AS**

**Gene families**	**Annotated genes in *Physcomitrella *** **( *n *)**	**Genes alternatively spliced under red or blue light ( *n *)**	**AS types**
*NPH3*	26	7	IR; AltA
*HY5/HYH*	2	2	IR; AltA; ES
*PIFs*	4	4	IR; AltD; AltA
*BBX22*	4	1	IR
*ELF3*	3	1	IR
*COP1*	9	6	IR; AltA
*CSN1-8*	13	10	IR; AltA
*DET1*	3	3	IR; AltD; AltA
*DDB1*	2	2	IR

**Figure 5:**
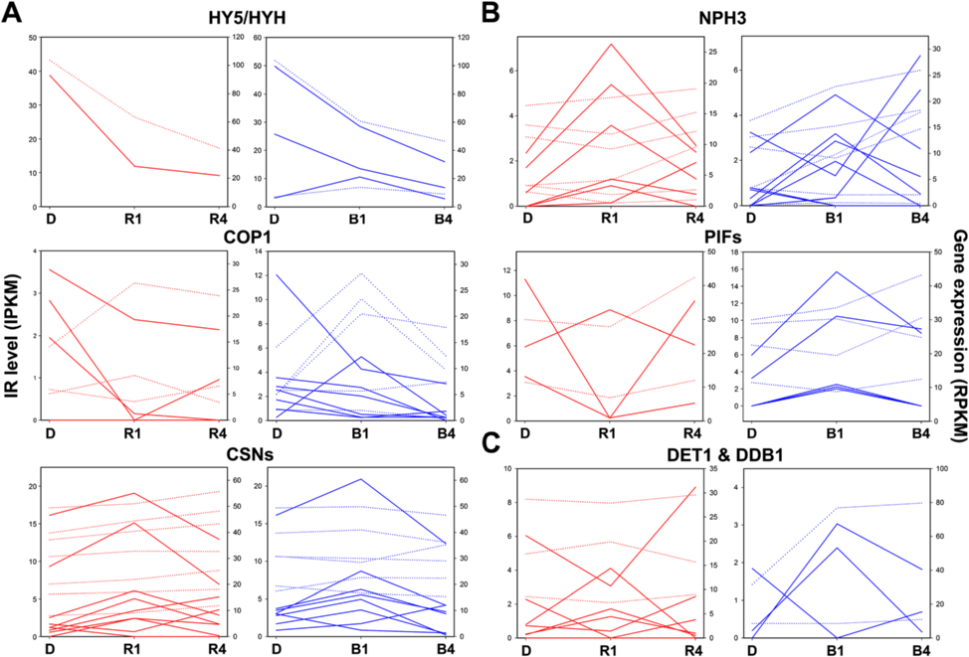
**IR of light signaling genes in response to red and blue light.** Expression profiles of red (left) and blue (right) light-regulated IR isoforms and corresponding genes of light signaling genes are shown. IPKM of IR transcripts (solid line) and RPKM of corresponding genes (dashed line) in dark-grown (D), 1-h light-treated (R1 and B1), 4-h light-treated (R4 and B4) samples were calculated and plotted. Events were arranged according to IR patterns of the gene families under red and blue lights. **(A)** Gene families showing a relatively consistent pattern among members under both red and blue lights. **(B)** Gene families showing a diverse pattern among members under their acting light conditions. **(C)** Gene families showing a diverse pattern among members under both light conditions. Note that some of the IR events occurred in transcripts of the same gene, therefore having the identical RPKM as overlapping dashed lines.

We next analyzed the patterns of light-regulated AS for these genes. Since all 36 genes underwent light-regulated IR, we therefore calculated IPKM and RPKM for the IR events occurred in these genes and validated by RT-PCR (Figure 5 and Additional file 1: Figure S6). Although IR patterns showed variations among these gene families, they can be divided into three groups. The first group of gene families including *HY5/HYH*, *COP1*, and *CSNs*, which encode key regulators in the downstream of both red- and blue-light signaling pathways, showed relatively consistent IR repression or induction after exposing 1 h of red and blue light (Figure 5A). Enhancing intron splicing in *HY5/HYH* transcripts could allow the production of more functional HY5/HYH, the positive regulator, to enhance photomorphogenesis responses. Meanwhile, immediate inhibition of intron splicing by light for CSNs, the photomorphogenesis repressor, then may temporarily abolish degradation of light signaling factors [26]. The result in COP1 family is elusive because IR repression of *COP1* transcripts potentially generates more E3 ligase for degrading light signaling regulators [25]. One explanation is COP1 may be required to degrade dark-accumulated photomorphogenesis repressors in the early stage of light exposure. It is also possible that COP1 in non-vascular plants have different functions.

The second group of transcripts from *NPH3* and *PIFs* genes showed diverse patterns under their acting light conditions, that is, *NPH3* under blue light and *PIFs* under red light, but rapid IR induction under inactive light conditions (Figure 5B). IR regulation in members of each gene family may promote differential activities of these factors under their acting wavelengths. *DET1* and *DDB1* represent the third group, which then showed diverse patterns under both red and blue lights (Figure 5C). In summary, our data indicate that light regulates AS of light signaling gene transcripts encoding central regulators downstream of both light signaling pathways as well as factors involved in specific light responses. The regulation could, at least partially, affect the abundance of light signaling factors and impact on the photomorphogenic control.

### *HYH* transcripts show complex but light-regulated AS in 5′ untranslated region

**Figure 6:**
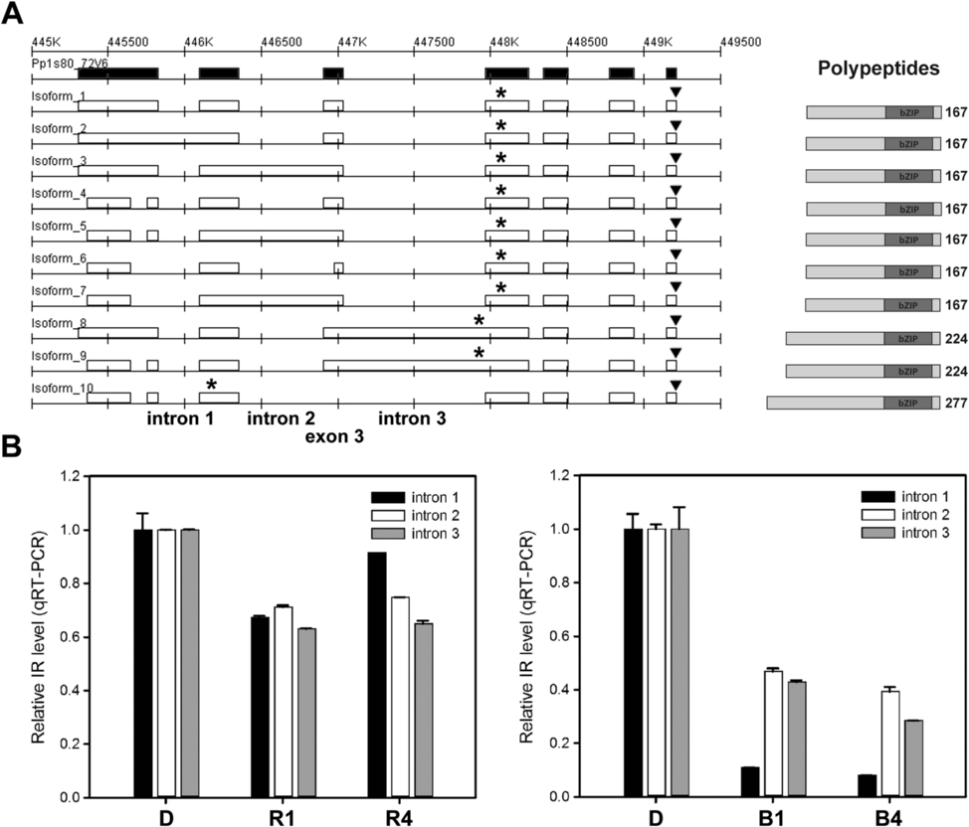
**AS events in *PpHYH2 *transcripts. (A)** Revised gene models of the *PpHYH2* locus. Expressed exonic regions (black) and putative isoforms are shown. Asterisks over each isoform indicate the start codons, and triangles represent the stop codons. Polypeptides encoded by the alternatively spliced isoforms were shown on the right. Sizes of the polypeptides are shown in the C-terminal end. Gray box, bZIP DNA-binding domain. **(B)** qRT-PCR analysis of relative expression of *PpHYH2* IR isoforms in pooled RNA from dark-grown (D), 1-h light-treated (R1 and B1), 4-h light-treated (R4 and B4) samples tested in triplicate with primer sets designed for *PpHYH2* IR isoforms, total transcripts of the corresponding genes, and *PpACT2* (see Additional file 7).

Among light signaling genes with light-regulated AS, *HYH* showed unique AS pattern that IR was repressed by both red and blue light. HYH interacts with HY5 and converges red- and blue-light signaling pathways for regulating transcription of light-responsive genes [41]. AS regulation occurring in *HYH* transcripts could have a strong effect on photomorphogenic gene regulation. In *Physcomitrella*, HY5 and HYH are annotated as HY5 Homolog 1 and 2 (PpHYH1 and PpHYH2). According to our mapping result, Sanger sequencing of RT-PCR products, and previous version of annotation, we revised the exon-intron structure for the *PpHYH2* gene. New gene models mainly encode 10 isoforms with complex IR and ES in the long 5′ untranslated region (5′UTR) (Figure 6A and Additional file 1: Figure S4). All three introns in 5′UTR are retained individually. Most of the IR isoforms can be translated into a 167 amino-acid protein with a bZIP DNA binding domain (DBD) in the C-terminal half, except the retention of intron 3 (isoform 8 and 9) or skipping of exon 3 (isoform 10) results in an extended N terminus of PpHYH2 protein. Although based on the sequence prediction all of the alternatively spliced transcripts of *PpHYH2* can produce a polypeptide with a bZIP DBD, IR in the 5′UTR potentially generates upstream open reading frames (uORFs). uORFs usually play a negative role in affecting translation of the downstream primary ORF [42]. IR in *PpHYH2* 5′UTR may increase the number of uORFs that could repress gene expression.

To validate the IR level of *PpHYH2* transcripts, we performed qRT-PCR. Primer pairs were designed to amplify the retained introns to determine IR level and constitutively-expression regions for total transcripts. Data showed that IR in *PpHYH2* 5′UTR were significantly repressed by red and blue light (Figure 6B). Blue light had a strong effect on the IR inhibition. This result suggests that intron splicing of *PpHYH2* transcripts is enhanced by light, which may shorten the *PpHYH2* 5′UTR and decrease the chance of uORF formation. This could increase the translation efficiency of the primary ORF and produce more functional HYH for positively regulating photomorphological gene transcription under the light.

### AltD and AltA are also regulated by light

Besides IR, AltD and AltA are also frequently found in plants. In *Physcomitrella*, we also observed large quantity of AltDA, AltD, and AltA events significantly affected by light (Figure 1). We again performed GO analysis to identify over-represented functions for these events. Red and blue light-responsive AltDA, AltD, and AltA events were pooled for functional enrichment analysis (see Additional file 1: Table S3). GO terms over-represented in red- and blue-light data were overall similar, except chloroplast-related terms were highly enriched from the blue light-regulated events. Of note, genes encoding nuclear proteins, kinases, and RNA polymerase II transcription factors or functioning in the ubiquitin-degradation catabolic process, RNA processing, and chromatin modification were enriched in the light-responsive AltD/AltA events. These gene products mostly act as regulators for gene expression, which implies that light may primarily and selectively modulate AS of key components involved in gene regulation. Expression of alternative transcripts in certain enriched GO terms was validated by high-resolution RT-PCR (see Additional file 1: Figure S4) [43]. In conclusion, our data reveal that light also regulates AltD and AltA in gene transcripts, especially for genes encoding regulatory factors in *Physcomitrella*.

### Motif analysis of IR regions reveals a repetitive GAA *cis* element enriched in exons

**Figure 7:**
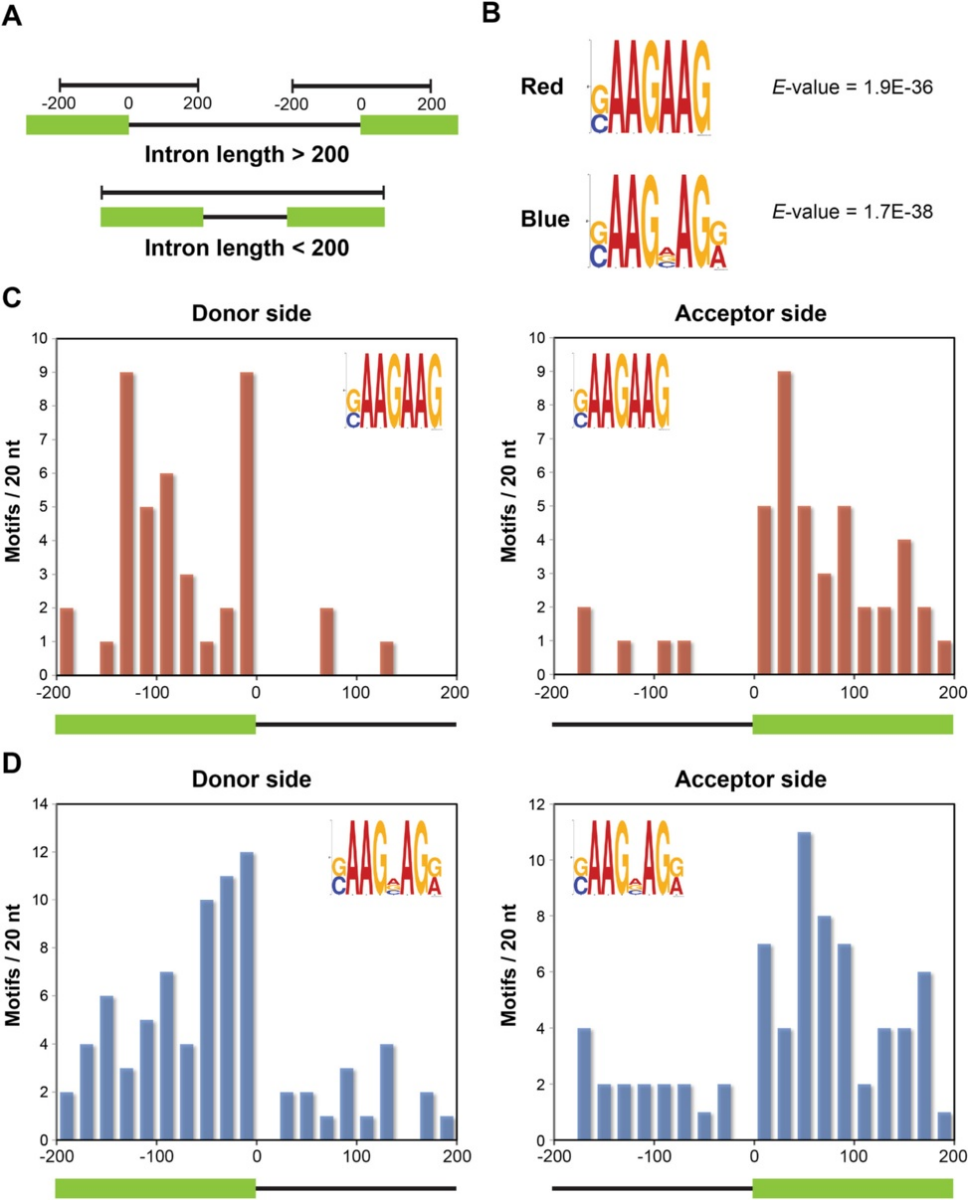
**Regulatory *cis *element in light-regulated IR regions. (A)** Illustration of sequence extraction for motif search. **(B)** The repetitive GAA motif enriched in red (top) and blue (bottom) light-regulated IR regions. The *E*-value indicating the statistical significance of the motif is shown on the right of the motif. Frequency distribution of the repetitive GAA motif in red **(C)** and blue **(D)** light-regulated IR regions are plotted. Frequencies of the motif were generated by counting the numbers of motif hits in windows of 20 nt near the donor and acceptor sites of light-regulated IR regions. Only input sequences extracted from donor and acceptor sites of the retained introns (longer than 200 nt) were used for motif counting. The green bar represents the exon and the solid line indicates the retained intron.

Regulatory *cis* elements presence in the AS regions can serve as binding sites for *trans* splicing factors to recruit the splicing machinery. In our data, a substantial number of events was highly light-regulated, which indicates that specific *cis* and *trans* components may also participate in the AS regulation. To examine whether the AS regions of light-regulated transcripts share common *cis* elements, we analyzed the light-regulated IR events using the MEME suite for motif search [44]. Three datasets were generated for motif analysis: sample, reference, and control datasets. To obtain the sample dataset, we extracted 200 nt of upstream and downstream flanking sequences from both 5′ and and 3′ splice sites of the top 500 retained introns that were responsive to red and blue light, respectively (Figure 7A). For introns shorter than 200 nt, the whole intron and adjacent exons were included. To generate the reference dataset, sequences in sample dataset were randomly shuffled. For the control dataset, sequences were extracted similar to that for the sample dataset but from 500 randomly selected IR events that were not statistically significant. We searched for conserved motifs from 7 to 12 mers. To further confirm the identified motifs preferentially located in the light-regulated IR regions, we compared the occurrence of the motif in the sample dataset to that in the control dataset based on the enrichment level with a *P* value of 0.001 by Fisher’s exact test.

In the red-light data, motif search yielded a highly conserved 7-nt sequence, [GC]AAGAAG, over-represented in red light-regulated IR regions, with an *E*-value of 1.9E-36 (Figure 7B, upper logo). Interestingly, the motif identified from blue-light-regulated IR regions, [GC]AAG[AGC]AG[GA], shared a similar pattern with the red-light motif. Although several nucleotides on this element are less conserved, it can be defined as a purine-rich motif with a repetitive GAA sequence. We also examined the distribution of the repetitive GAA motifs near donor and acceptor sites of retained introns. Frequency distribution of both red- and blue-light motifs were plotted by counting the number of motif hits in windows of 20 nt. Most of the motif hits were located in adjacent exons of the retained introns, in general showing a higher frequency close to the splice sites (Figure 7C and D). This evidence further supports that the repetitive GAA *cis* element is an exonic splicing regulator that functions in light-regulated IR. The similar motif pattern in both red- and blue-light data reveals that the repetitive GAA element may commonly function as a binding site for splicing regulators in red and blue light-mediated splicing regulation.

### Phytochromes primarily participate in splicing regulation

**Figure 8:**
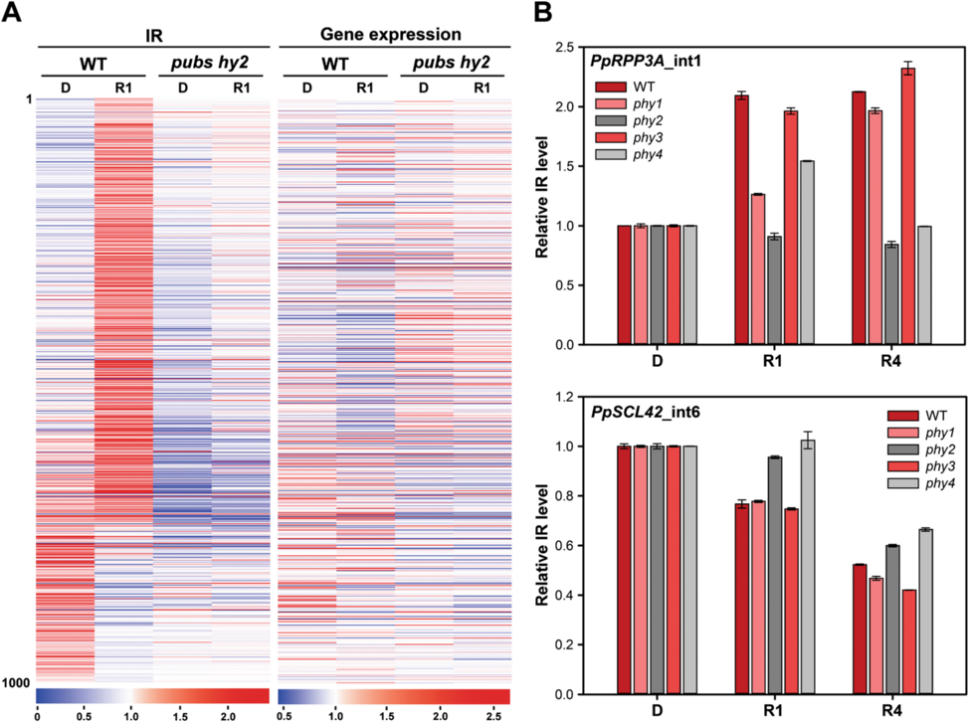
**Red light induction of IR is misregulated in the phytochrome-deficient and -knockout mutants. (A)** Expression profiles of top 1,000 red light-regulated IR isoforms and total transcripts of corresponding genes in wild type (WT) and *pubs hy2* mutant are shown. IR level (IR) and corresponding gene expression level (Gene expression) for the top 1,000 IR events were normalized by mean across WT and mutant samples. Heat maps of these events in dark-grown (D) and 1-h red light-treated (R1) samples are generated. Events were arranged according to the fold-change of IR level comparing 1-h red light-treated to dark-grown samples in WT. The scale of expression is shown in the bottom. **(B)** IR of *PpRPP3A* and *PpSCL42* in WT and phytochrome-knockout mutants. Quantitative RT-PCR analysis of IR level in samples from WT and four phytochrome-knockout mutants (*phy1*, *phy2*, *phy3*, *phy4*) grown under dark (D), 1-h (R1), and 4-h (R4) of red light. Both genes were tested in triplicate with primer sets designed for IR isoforms, total transcripts of the corresponding genes, and *PpACT2* (see Additional file 7).

Photomorphogenic gene regulation mainly relies on light-sensing photoreceptors. Our data showed that AS in *Physcomitrella* is immediately affected within 1 h of red- and blue-light exposure. Such early response is likely mediated directly by photoreceptors. To determine whether photoreceptors are involved in the light regulation of AS, we compared IR pattern in wild type (WT) and a mutant that is defective in red light sensing. In a previous study, we generated a *Physcomitrella* knockout mutant with gene disruptions at loci encoding the phytochromobilin synthase (HY2) and phycourobilin synthase (PUBS). The *pubs hy2* double mutant is defective in biosynthesis of phytochrome chromophore and characterized to be a phytochrome-deficient mutant [34]. RNA-seq data previously generated for the *pubs hy2* double mutant were further analyzed. In WT, more than 70% of the top 1,000 IR events significantly affected after 1-h red light showed the pattern of light induction (Figure 8A, left panel). However, retention of these introns was mostly decreased in the *pubs hy2* double mutant after 1 h of red light exposure. On the other hand, those top 1,000 IR events repressed by red light in WT (bottom part of the list) also became largely unresponsive in the phytochrome-deficient mutant. Corresponding gene expression level for the top 1,000 IR events showed that light-mediated changes of IR level are not associated with transcriptional activity (Figure 8A, right panel). Overall, IR was rapidly induced by light in WT but misregulated globally in the *pubs hy2* double mutant, suggesting the involvement of phytochromes in splicing regulation.

Seven phytochromes are found in *Physcomitrella* and physiological functions for several of them have been characterized [45-48]. To further determine the role of individual phytochromes in regulation of AS, we generated knockout mutants for PpPHY1, PpPHY2, PpPHY3, and PpPHY4 by gene targeting and designated them as *phy1*, *phy2*, *phy3*, and *phy4* (see Additional file 1: Figure S7). IR level of two genes encoding the ribosomal protein PpRPP3A and SR protein PpSCL42 were measured by qRT-PCR in WT and four phytochrome knockout mutants grown under the dark, 1 h, and 4 h of red light (Figure 8B and Additional file 1: Figure S5). Among four phytochrome-knockout mutants, we found *phy1* and *phy3* mutants still behave similarly as the WT with increased IR level for *PpRPP3A* and decreased for *PpSCL42*. However, IR of both genes was less responsive to red light in *phy2* and *phy4* mutants. Interestingly, PpPHY1 and PpPHY3 have been phylogenetically clustered and physiologically characterized as the phyA-type members. The rest of *Physcomitrella* phytochromes belong to the phyB type [48,49]. Our results suggest phyB-type phytochromes play more dominated roles under red light condition for IR regulation. Because the single mutants still showed partial light responsiveness, we propose that multiple phyB-type phytochromes are involved in the regulatory process. In conclusion, the photoreceptors, at least phytochromes, are the determinants for regulating AS.

## Discussion

### AS is another level of gene regulation for light responses in plants

Regulation of gene expression can occur at different levels to control the abundance of functional gene products for cellular differentiation and morphogenesis. In plant photomorphogenesis, regulation at the chromatin, transcription, translation, and post-translational levels have been reported and well characterized [13,14,17-19,24-26]. In this study, we performed a transcriptome-wide study to suggest that global gene regulation during light exposure also takes place at the mRNA splicing step. By taking advantage of next-generation sequencing and bioinformatics, we observed changes in AS patterns in response to light in *Physcomitrella* protonema. This finding indicates that AS is also abundant in non-vascular plants and that it can be regulated by light to alter the transcriptome for photomorphogenesis. We also observed immediate induction of IR when dark-grown cells exposed to the light (Figure 3). Such IR induction potentially generates unproductive transcripts or truncated proteins, thus resulting in the suppression of cellular processes. This process could be a safety procedure to temporarily attenuate cell growth for preventing light damage. In the later stage of light exposure, IR then becomes moderate and can promote vegetative growth under light. Expression of regulators in RNA splicing and light signaling is also modulated at the mRNA splicing step for splicing control and light-mediated gene expression.

To attenuate cell growth, an efficient way is to control key biological processes. Our data indicate that immediate IR induction by light occurs largely in transcripts of photosynthesis and ribosomal proteins (Figure 3). Both are important for energy and protein production in plant cells. Photosynthesis activity needs to be tightly controlled in response to light conditions. When gene transcription is not yet regulated by light signaling during the initial stage of light exposure, immediate induction of IR on photosynthesis gene transcripts could transiently decrease the production of photosynthetic proteins for function. Light repression of intron splicing on transcripts encoding ribosomal proteins could then globally inhibit translation in the cell or even play a role in specific translational control.

Besides metabolic processes, AS occurred in regulatory genes are also modulated by light. Light-induced intron splicing for SR protein gene transcripts suggest that although these splicing factors may not be primarily involved the immediate regulation of AS during light exposure, they could be important for subsequent splicing regulation in response to light conditions. Differential IR regulation under red and blue light for these genes further support that splicing factors may function differently under specific light conditions. We also observed significant amount of light signaling genes are alternatively spliced in response to light (Figure 5 and Table 2). AS of these key regulators could have a strong impact on photomorphogenic control for plants to increase transcriptome plasticity upon light exposure.

### Involvement of photoreceptors for splicing regulation

In our study, two wavelengths of light were used to determine the light-dependent AS events. In the analysis of light-regulated IR, although the number of IR events responding to red and blue light were similar, events commonly regulated by both red and blue light are minor (Figure 1B). A large proportion of IR events were specifically regulated only by red or blue light. These observations support that red and blue light-dependent mechanisms may exist for regulating IR of different transcripts. Our hypothesis is further strengthened by the results of IR analysis in phytochrome-deficient and knockout mutants (Figure 8). Misregulation occurred in mutants for those light-responsive IR events reveal that phytochromes are primarily involved in the regulatory process. Because cryptogam phytochromes can also accumulate in the nucleus in a light-dependent manner [48], we therefore propose that red light-promoted splicing regulation in *Physcomitrella* is primarily mediated by light-activated, nuclear-localized phytochromes. Our data also indicate that phyB-type phytochromes play the dominant role in red light-regulated AS (Figure 8B), suggesting that wavelength-dependent mechanisms for splicing regulation are present in plants. Because the immediate, light-dependent AS regulation also occurs under the blue light condition, we believe blue-light photoreceptors are also required for the splicing regulation. Further analysis will be needed to support this hypothesis.

A similar pattern of IR induction and regulatory elements were found in both red and blue light-regulated IR events (Figure 7). A possible explanation is that red and blue light may affect the same splicing component(s) for regulation, but there are red and blue light-dependent mechanisms to determine transcript specificity of AS. One potential mechanism is through light-mediated chromatin modification as described below. Studies for the role of photoreceptors in AS regulation is undergoing.

### AS in plants possesses transcript specificity in response to environmental factors

AS is commonly found in eukaryotic organisms and plays a major role in increasing overall transcriptome complexity and proteomic diversity. However, our data showed that light-regulated AS is predominant in transcripts involved in specific biological processes (Table 1). A question is how the splicing machinery differentiates AS transcripts. One possibility for transcript selection is the co-transcriptional control mediated by chromatin modification. Accumulating studies in AS regulation have provided new views of the co-transcriptional splicing mediated by epigenetic control [50,51]. Histone modification can affect AS by influencing the recruitment of splicing regulators to chromatin [52-54]. In plants, chromatin remodeling associated with light signaling is known for transcriptional regulation [13]. Photosynthesis genes have been shown as the targets of light-mediated chromatin modification during de-etiolation [55-59]. Photoreceptor systems are possibly involved in light control for histone modifications [55]. In our analysis, AS of photosynthesis genes were also regulated in a light-dependent manner. We also found that AS occurs actively in the *PpHYH2* gene (Figure 6). Interestingly, the *Arabidopsis HY5* and *HYH* loci revealed significant light-dependent histone modification [56]. These results reveal a possible link between light-dependent regulation of AS and chromatin modification in plants. Further investigation will be required to support this hypothesis.

### Sequence-specific control of light-responsive AS

Splicing regulators are also potential candidates involved in transcript-specific AS in response to light. In the light-regulated IR regions, we identified a purine-rich, repetitive GAA motif in exonic regions that potentially function as a regulatory element on IR transcripts. The repetitive GAA motif was enriched in light-regulated IR regions, which suggests its involvement in splicing regulation under light. This purine-rich sequence with GAA repeats on pre-mRNA is a splicing enhancer in mammalian systems [60-62]. An AG-rich exonic *cis* element has been shown capable of promoting downstream donor site recognition in plants [63]. In *Arabidopsis*, the repetitive GAA sequence was proposed as an exonic splicing enhancer (ESE) [64] (Arabidopsis 2010 project, [65]). This purine-rich splicing enhancer is recognized by SR proteins in animals [66,67]. A recent study has also shown purine-rich GAAG repeats in the intronic region function as the binding site for SCL33 protein in *Arabidopsis*[68]. Plant SR proteins may recognize the purine-rich sequence to regulate intron splicing. In our study, the GAA motif we identified is present in the IR regions mostly induced by light, which suggests this motif either functions as a silencer or losses enhancer activity during light-mediated splicing regulation for specific transcripts.

The status of splicing regulators could be the determinant for splicing regulation. For example, subcellular localization of SR proteins can modulate the splicing activity, because nuclear localization of splicing regulators is dynamic [69]. Moreover, post-translational modification can also affect the activity of SR proteins. The arginine/serine (RS) domain of SR proteins is the prominent target for phosphorylation [70,71]. Likely, light-mediated phosphorylation/dephosphorylation of specific splicing regulators allows the splicing machinery to differentiate transcripts for AS and control splice site recognition. We propose that unknown splicing regulators are involved in regulating IR of specific transcripts in response to light. How the purine-rich *cis* element regulates IR in plants requires the identification and characterization of its *trans* binding factors.

### Evolution of light-mediated transcriptional and post-transcriptional regulation

Mosses are ancient non-vascular plants diverged early in land plant evolution. Therefore, the evolutionary position of mosses in the intermediate group of green lineage reveals its importance in the study of conserved biological processes for photosynthetic organisms. In the previous study, we have observed that light-mediated transcriptional regulation is conserved at least in part among land plants. The occurrence of AS in the moss is also comparable to that in higher plants. Nevertheless, the finding of abundant AS events in moss protonema cells strongly supports that AS patterns could have diversified already during land colonization of aquatic photosynthetic organisms, and retained in higher plants.

## Conclusions

In this study, we used RNA-seq to study light-regulated gene expression at the post-transcriptional level in *Physcomitrella*. We have provided the first genome-wide analysis to show that alternative splicing is regulated by light in plants. Evidence from this study suggests that the mRNA splicing step can be rapidly modulated by light to control AS of transcripts involved in specific functions. Such regulation is important for light responses in plants. We have also identified a regulatory *cis* element involved in light-mediated splicing regulation. This result may allow identifying the potential RNA-binding proteins or splicing regulators involved in the light-mediated AS regulation. We also found that IR was rapidly induced by light but misregulated in moss mutants defective in red light sensing phytochromes, suggesting that photoreceptors primarily participate in regulating AS in plants. Our study is the first to report a transcriptome-wide analysis for AS in non-vascular plants. The results demonstrate that patterns of AS are evolutionarily conserved in land plants. Although the current approach is unable to reveal the mechanism of light-regulated AS, it provides many new directions to pursue. We hope that further investigation on the regulatory mechanism of mRNA splicing can shed light on studies of gene regulation in plants in response to environmental factors.

## Materials and methods

### Plant materials and growth conditions

Protonemata of *Physcomitrella patens* subsp. patens were grown on solid Knop’s medium or solid BCDAT medium. Spores were germinated on solid Knop’s medium supplemented with 10 mM CaCl_2_ and overlaid with cellophane for approximately 14 to 16 days. Plants were cultured at 25°C under continuous white light (80-100 μmol m^-2^ s^-1^).

### Light treatment, RNA isolation, and mRNA sequencing

The 10-day-old protonemata of the WT were grown in the dark for 3 days followed by 1- and 4-h red-light treatment (660 nm LED, 5 μmol m^-2^ s^-1^) at 25°C as previously reported [34]. For blue-light treatment, a light intensity similar to that in a previous study was used (472 nm LED, 17 μmol m^-2^ s^-1^) [72]. Dark-grown protonema cells were collected as the dark control. RNA isolation and cDNA library preparation were performed as described previously [34]. Sequencing was performed on the Hiseq 2000 at Yourgene Bioscience, Taiwan. On average, 39 million 100-nt paired-end reads for each sample were obtained.

### Read mapping to the reference genome

Sequence reads were mapped to the *Physcomitrella patens* genome V1.6 ([73], data can also be found in JGI, [74]) by use of the BLAT program [35,75]. Reads per kilobase of exon model per million mapped reads (RPKMs) computation involved use of the RACKJ package ([76]) with a similar algorithm as described previously [36].

### Prediction of AS

RACKJ was used to compute the following read counts (mapping by BLAT) and separated into several tables: (1) for every exon; (2) for every intron; (3) for every exon-pair that were mapped by splice reads; and (4) for every exon-pair plus junction shifts that were supported by spliced reads. AS events were then computed accordingly. The fourth table records potential AltA/AltD events. From the aforementioned tables, perl scripts in RACKJ package ([76]) were used for detecting AS (IR, ES, and AltA/AltD) events for red- and blue-light samples [37]. Chi-square values for goodness-of-fit were computed by comparing read counts supporting an AS event (that is, intronic read counts, exon-skipped read counts, and alternative donor/acceptor read counts) and read counts not supporting the AS event (for example, read counts of corresponding gene exons, read counts of neighboring exons, read counts involving a skipped exon, and read counts of the same exon-pair but not of the same AltA/AltD events). In so doing, we then computed corresponding *P* values by chi-square distribution using Microsoft Excel (Microsoft, Redmond, WA, USA).

### Gene ontology analysis

Functional enrichment analysis involved the Gene Ontology Browsing Utility (GOBU) with MultiView ([77]) [78]. *Physcomitrella* gene sequences were first subjected to the Bio301 system to generate GO annotations for all transcripts [79]. The Bio301 system predicts functions by blasting against UniProt and RefSeq databases and by InterProt domain prediction to generate the reference GO annotation set. *P* values for over-represented GO terms were computed by comparing the differentially regulated gene set to the reference GO annotation set using the ‘elim’ method. The elim method is a Java implementation of the TopGO ‘elim’ algorithm [80]. For generating heat maps, relative IR and gene expression level were plotted by use of Java Treeview [81].

### Analysis of *cis* regulatory elements

Motif search involved the MEME suite [44]. An extra run of MAST, a member program of MEME, with the option -hit_list was performed for searching motif sites on promoter or AS regions. Note that every predicted motif site in the MAST output is associated with a *P* value for similarity. To compute the preference of a given motif under a certain MAST *P* value threshold, Fisher’s exact test was performed with the following four numbers: (1) light-regulated promoter or AS regions hit (that is, input sequences with at least one motif occurrence); (2) randomly selected promoter or AS regions hit; (3) light-regulated promoter or AS regions not hit; and (4) randomly selected promoter or AS regions not hit. A motif that is preferentially present in the light-regulated promoter or AS regions would show a significant *P* value.

### Quantitative RT-PCR analysis

The cDNA synthesis was performed with 4.5 μg of total RNA, an oligo(dT), and SuperScript III RT kit (Invitrogen, USA). qRT-PCR analysis involved the 7500 Real Time PCR System (Applied Biosystems, USA) with the Power SYBR Green PCR Master Mix (Applied Biosystems, USA). Primers designed by Primer Express Software v3.0 (Applied Biosystems, USA) are in Additional file 7. qRT-PCR reactions were in triplicate. *PpACT2* (*Pp1s198_157V6*) was used as an internal control for normalization in qRT-PCR.

### High-resolution RT-PCR

High-resolution RT-PCR was performed as described previously with minor modification [43]. PCR involved AS-specific primers (Additional file 7). One strand of the primer pair was labeled with the fluorescent dye 5-carboxyfluorescein (5′-FAM). RT-PCR product (1 μL) was diluted into 10 μL mixture of Hi-Di formamide and GeneScan 600 LIZ internal size standard (90:1 ratio) (Applied Biosystems, USA). DNA fragments were separated on an ABI3730 DNA Analyzer (Applied Biosystems, USA) with three technical repeats and then analyzed by use of Peak Scanner v1.0. RT-PCR products from corresponding AS isoforms were identified with expected sizes. The percentage of each AS isoform was calculated as the fluorescent peak areas of AS transcripts divided by the sum of all detected transcripts. In comparing results from three samples by ANOVA, *P* values were generated.

### Data deposition

RNA-seq data from this publication have been submitted to the National Center for Biotechnology Information-Sequence Read Archive database (SRA, [82]) and assigned the identifier SRX252526.

## Competing interests

The authors declare that they have no competing interests.

## Authors’ contributions

S-LT designed the research. H-PW, Y-sS, Y-RC, H-CC, C-CW, and W-DL performed the research. H-PW, Y-sS, and S-LT analyzed the data. S-LT wrote the paper. All authors read and approved the final manuscript.

## Supplementary Material

Additional file 1 Contains Supplementary Figures S1 to S5 and Supplementary Tables S1 to S3. Click here for file

Additional file 2 Tables listing all and statistically significant IR, AltDA, AltA, AltD, and ES events found in red-light data. Click here for file

Additional file 3 Tables listing all and statistically significant IR, AltDA, AltA, AltD, and ES events found in blue-light data. Click here for file

Additional file 7 Table listing all primers used in this study. Click here for file

Additional file 4 Tables listing splicing-related genes found in statistically significant IR events of red-light data. Click here for file

Additional file 5 Tables listing splicing-related genes found in statistically significant IR events of blue-light data. Click here for file

Additional file 6 Tables listing light signaling genes found in statistically significant IR events of red- and blue-light data. Click here for file

## References

[B1] Maniatis T Mechanisms of alternative pre-mRNA splicing Science 1991 251 33 34 10.1126/science.1824726 1824726

[B2] Reddy AS Alternative splicing of pre-messenger RNAs in plants in the genomic era Annu Rev Plant Biol 2007 58 267 294 10.1146/annurev.arplant.58.032806.103754 17222076

[B3] Black DL Mechanisms of alternative pre-messenger RNA splicing Annu Rev Biochem 2003 72 291 336 10.1146/annurev.biochem.72.121801.161720 12626338

[B4] Syed NH Kalyna M Marquez Y Barta A Brown JWS Alternative splicing in plants - coming of age Trends Plant Sci 2012 17 616 623 10.1016/j.tplants.2012.06.001 22743067 PMC3466422

[B5] Filichkin SA Priest HD Givan SA Shen R Bryant DW Fox SE Wong W-K Mockler TC Genome-wide mapping of alternative splicing in *Arabidopsis thaliana* Genome Res 2010 20 45 58 10.1101/gr.093302.109 19858364 PMC2798830

[B6] Marquez Y Brown JWS Simpson C Barta A Kalyna M Transcriptome survey reveals increased complexity of the alternative splicing landscape in *Arabidopsis* Genome Res 2012 22 1184 1195 10.1101/gr.134106.111 22391557 PMC3371709

[B7] Zhang G Guo G Hu X Zhang Y Li Q Li R Zhuang R Lu Z He Z Fang X Chen L Tian W Tao Y Kristiansen K Zhang X Li S Yang H Wang J Deep RNA sequencing at single base-pair resolution reveals high complexity of the rice transcriptome Genome Res 2010 20 646 654 10.1101/gr.100677.109 20305017 PMC2860166

[B8] Chang Y-F Imam JS Wilkinson MF The nonsense-mediated decay RNA surveillance pathway Ann Rev Biochem 2007 76 51 74 10.1146/annurev.biochem.76.050106.093909 17352659

[B9] Kalyna M Simpson CG Syed NH Lewandowska D Marquez Y Kusenda B Marshall J Fuller J Cardle L McNicol J Dinh HQ Barta A Brown JW Alternative splicing and nonsense-mediated decay modulate expression of important regulatory genes in *Arabidopsis* Nucleic Acids Res 2012 40 2454 2469 10.1093/nar/gkr932 22127866 PMC3315328

[B10] Briggs WR Spudich JA (eds): Handbook of Photosensory Receptors 2005 Wiley-VCH: Weinheim

[B11] Kami C Lorrain S Hornitschek P Fankhauser C Marja CPT Light-regulated plant growth and development Current Topics in Developmental Biology, Volume 91 2010 Waltham, MA: Academic Press 29 66 10.1016/S0070-2153(10)91002-8 20705178

[B12] Huq E Quail PH Briggs WR, Spudich JA Phytochrome signaling Handbook of Photosensory Receptors 2005 Weinheim: Wiley-VCH 151 170

[B13] Fisher AJ Franklin KA Chromatin remodelling in plant light signalling Physiol Plant 2011 142 305 313 10.1111/j.1399-3054.2011.01476.x 21457270

[B14] Li J Terzaghi W Deng XW Genomic basis for light control of plant development Protein Cell 2012 3 106 116 10.1007/s13238-012-2016-7 22426979 PMC4875414

[B15] Schroeder DF Gahrtz M Maxwell BB Cook RK Kan JM Alonso JM Ecker JR Chory J De-etiolated 1 and damaged DNA binding protein 1 interact to regulate *Arabidopsis* photomorphogenesis Curr Biol 2002 12 1462 1472 10.1016/S0960-9822(02)01106-5 12225661

[B16] Casal JJ Yanovsky MJ Regulation of gene expression by light Int J Dev Biol 2005 49 501 511 10.1387/ijdb.051973jc 16096960

[B17] Jenkins GI Signal transduction in responses to UV-B radiation Annu Rev Plant Biol 2009 60 407 431 10.1146/annurev.arplant.59.032607.092953 19400728

[B18] Chen M Chory J Phytochrome signaling mechanisms and the control of plant development Trends Cell Biol 2011 21 664 671 10.1016/j.tcb.2011.07.002 21852137 PMC3205231

[B19] Leivar P Quail PH PIFs: pivotal components in a cellular signaling hub Trends Plant Sci 2011 16 19 28 20833098 10.1016/j.tplants.2010.08.003 PMC3019249

[B20] Petracek ME Dickey LF Huber SC Thompson WF Light-regulated changes in abundance and polyribosome association of ferredoxin mRNA are dependent on photosynthesis Plant Cell 1997 9 2291 2300 9437868 10.1105/tpc.9.12.2291 PMC157075

[B21] Helliwell CA Webster CI Gray JC Light-regulated expression of the pea plastocyanin gene is mediated by elements within the transcribed region of the gene Plant J 1997 12 499 506 10.1046/j.1365-313X.1997.d01-6.x 9351238

[B22] Dickey LF Petracek ME Nguyen TT Hansen ER Thompson WF Light regulation of Fed-1 mRNA requires an element in the 5′ untranslated region and correlates with differential polyribosome association Plant Cell 1998 10 475 484 9501119 10.1105/tpc.10.3.475 PMC143995

[B23] McKim SM Durnford DG Translational regulation of light-harvesting complex expression during photoacclimation to high-light in *Chlamydomonas reinhardtii* Plant Physiol Biochem 2006 44 857 865 10.1016/j.plaphy.2006.10.018 17097295

[B24] Liu M-J Wu S-H Chen H-M Wu S-H Widespread translational control contributes to the regulation of *Arabidopsis* photomorphogenesis Mol Syst Biol 2012 8 566 22252389 10.1038/msb.2011.97 PMC3296358

[B25] Lau OS Deng XW The photomorphogenic repressors COP1 and DET1: 20 years later Trends Plant Sci 2012 17 584 593 10.1016/j.tplants.2012.05.004 22705257

[B26] Wei N Deng XW The COP9 signalsome Ann Rev Cell Dev Biol 2003 19 261 286 10.1146/annurev.cellbio.19.111301.112449 14570571

[B27] Mastrangelo AM Marone D Laidò G De Leonardis AM De Vita P Alternative splicing: Enhancing ability to cope with stress via transcriptome plasticity Plant Sci 2012 185–186 40 49 10.1016/j.plantsci.2011.09.006 22325865

[B28] Mano S Hayashi M Nishimura M A leaf-peroxisomal protein, hydroxypyruvate reductase, is produced by light-regulated alternative splicing Cell Biochem Biophys 2000 32 147 154 10.1385/CBB:32:1-3:147 11330041

[B29] Yoshimura K Mori T Yokoyama K Koike Y Tanabe N Sato N Takahashi H Maruta T Shigeoka S Identification of alternative splicing events regulated by an *Arabidopsis* serine/arginine-like protein, atSR45a, in response to high-light stress using a tiling array Plant Cell Physiol 2011 52 1786 1805 10.1093/pcp/pcr115 21862516

[B30] Seo PJ Park MJ Lim MH Kim SG Lee M Baldwin IT Park CM A self-regulatory circuit of CIRCADIAN CLOCK-ASSOCIATED1 underlies the circadian clock regulation of temperature responses in *Arabidopsis* Plant Cell 2012 24 2427 2442 10.1105/tpc.112.098723 22715042 PMC3406914

[B31] Penfield S Josse EM Halliday KJ A role for an alternative splice variant of PIF6 in the control of *Arabidopsis* primary seed dormancy Plant Mol Biol 2010 73 89 95 10.1007/s11103-009-9571-1 19911288

[B32] Zhou DX Kim YJ Li YF Carol P Mache R COP1b, an isoform of COP1 generated by alternative splicing, has a negative effect on COP1 function in regulating light-dependent seedling development in *Arabidopsis* Mol Gen Genet 1998 257 387 391 10.1007/s004380050662 9529519

[B33] Shikata H Shibata M Ushijima T Nakashima M Kong SG Matsuoka K Lin C Matsushita T The RS domain of *Arabidopsis* splicing factor RRC1 is required for phytochrome B signal transduction Plant J 2012 70 727 738 10.1111/j.1365-313X.2012.04937.x 22324426

[B34] Chen Y-R Su Y-s Tu S-L Distinct phytochrome actions in nonvascular plants revealed by targeted inactivation of phytobilin biosynthesis Proc Natl Acad Sci USA 2012 109 8310 8315 10.1073/pnas.1201744109 22566621 PMC3361420

[B35] Zimmer AD Lang D Buchta K Rombauts S Nishiyama T Hasebe M Van de Peer Y Rensing SA Reski R Reannotation and extended community resources for the genome of the non-seed plant *Physcomitrella patens* provide insights into the evolution of plant gene structures and functions BMC Genomics 2013 14 498 10.1186/1471-2164-14-498 23879659 PMC3729371

[B36] Mortazavi A Williams BA McCue K Schaeffer L Wold B Mapping and quantifying mammalian transcriptomes by RNA-Seq Nat Meth 2008 5 621 628 10.1038/nmeth.1226 PMC13303166 18516045

[B37] Li W Lin W-D Ray P Lan P Schmidt W Genome-wide detection of condition-sensitive alternative splicing in *Arabidopsis* roots Plant Physiol 2013 162 1750 1763 10.1104/pp.113.217778 23735510 PMC3700675

[B38] Lan P Li W Lin W-D Santi S Schmidt W Mapping gene activity of *Arabidopsis* root hairs Genome Biol 2013 14 R67 10.1186/gb-2013-14-6-r67 23800126 PMC3707065

[B39] Wang BB Brendel V The ASRG database: identification and survey of *Arabidopsis thaliana* genes involved in pre-mRNA splicing Genome Biol 2004 5 R102 10.1186/gb-2004-5-12-r102 15575968 PMC545797

[B40] Reddy ASN Shad Ali G Plant serine/arginine-rich proteins: roles in precursor messenger RNA splicing, plant development, and stress responses Wiley Interdisciplinary Reviews: RNA 2011 2 875 889 10.1002/wrna.98 21766458

[B41] Holm M Ma L-G Qu L-J Deng X-W Two interacting bZIP proteins are direct targets of COP1-mediated control of light-dependent gene expression in *Arabidopsis* Gene Dev 2002 16 1247 1259 10.1101/gad.969702 12023303 PMC186273

[B42] Morris DR Geballe AP Upstream open reading frames as regulators of mRNA translation Mol Cell Biol 2000 20 8635 8642 10.1128/MCB.20.23.8635-8642.2000 11073965 PMC86464

[B43] Simpson CG Fuller J Maronova M Kalyna M Davidson D McNicol J Barta A Brown JW Monitoring changes in alternative precursor messenger RNA splicing in multiple gene transcripts Plant J 2008 53 1035 1048 18088312 10.1111/j.1365-313X.2007.03392.x

[B44] Bailey TL Boden M Buske FA Frith M Grant CE Clementi L Ren J Li WW Noble WS MEME Suite: tools for motif discovery and searching Nucl Acids Res 2009 37 W202 W208 10.1093/nar/gkp335 19458158 PMC2703892

[B45] Rensing SA Lang D Zimmer AD Terry A Salamov A Shapiro H Nishiyama T Perroud P-F Lindquist EA Kamisugi Y Tanahashi T Sakakibara K Fujita T Oishi K Shin-I T Kuroki Y Toyoda A Suzuki Y Hashimoto S-i Yamaguchi K Sugano S Kohara Y Fujiyama A Anterola A Aoki S Ashton N Barbazuk WB Barker E Bennetzen JL Blankenship R The *Physcomitrella* genome reveals evolutionary insights into the conquest of land by plants Science 2008 319 64 69 10.1126/science.1150646 18079367

[B46] Mittmann F Brucker G Zeidler M Repp A Abts T Hartmann E Hughes J Targeted knockout in *Physcomitrella* reveals direct actions of phytochrome in the cytoplasm Proc Natl Acad Sci USA 2004 101 13939 13944 10.1073/pnas.0403140101 15365180 PMC518857

[B47] Uenaka H Kadota A Functional analyses of the *Physcomitrella patens* phytochromes in regulating chloroplast avoidance movement Plant J 2007 51 1050 1061 10.1111/j.1365-313X.2007.03202.x 17662030

[B48] Possart A Hiltbrunner A An evolutionarily conserved signaling mechanism mediates far-red light responses in land plants Plant Cell 2013 25 102 114 10.1105/tpc.112.104331 23303916 PMC3584528

[B49] Mittmann F Dienstbach S Weisert A Forreiter C Analysis of the phytochrome gene family in Ceratodon purpureus by gene targeting reveals the primary phytochrome responsible for photo- and polarotropism Planta 2009 230 27 37 10.1007/s00425-009-0922-6 19330350

[B50] Luco RF Allo M Schor IE Kornblihtt AR Misteli T Epigenetics in alternative pre-mRNA slicing Cell 2011 144 16 26 10.1016/j.cell.2010.11.056 21215366 PMC3038581

[B51] Schwartz S Ast G Chromatin density and splicing destiny: on the cross-talk between chromatin structure and splicing EMBO J 2010 29 1629 1636 10.1038/emboj.2010.71 20407423 PMC2876972

[B52] Luco RF Pan Q Tominaga K Blencowe BJ Pereira-Smith OM Misteli T Regulation of alternative splicing by histone modifications Science 2010 327 996 1000 10.1126/science.1184208 20133523 PMC2913848

[B53] Pradeepa MM Sutherland HG Ule J Grimes GR Bickmore WA Psip1/Ledgf p52 binds methylated histone H3K36 and splicing factors and contributes to the regulation of alternative splicing PLoS Genet 2012 8 e1002717 10.1371/journal.pgen.1002717 22615581 PMC3355077

[B54] Moehle EA Ryan CJ Krogan NJ Kress TL Guthrie C The yeast SR-like protein Npl3 links chromatin modification to mRNA processing PLoS Genet 2012 8 29 10.1371/journal.pgen.1003101 PMC3510044 23209445

[B55] Guo L Zhou J Elling AA Charron JB Deng XW Histone modifications and expression of light-regulated genes in *Arabidopsis* are cooperatively influenced by changing light conditions Plant Physiol 2008 147 2070 2083 10.1104/pp.108.122929 18550682 PMC2492627

[B56] Charron JB He H Elling AA Deng XW Dynamic landscapes of four histone modifications during deetiolation in *Arabidopsis* Plant Cell 2009 21 3732 3748 10.1105/tpc.109.066845 20008096 PMC2814509

[B57] Chua YL Brown AP Gray JC Targeted histone acetylation and altered nuclease accessibility over short regions of the pea plastocyanin gene Plant Cell 2001 13 599 612 11251099 10.1105/tpc.13.3.599 PMC135505

[B58] Chua YL Watson LA Gray JC The transcriptional enhancer of the pea plastocyanin gene associates with the nuclear matrix and regulates gene expression through histone acetylation Plant Cell 2003 15 1468 1479 10.1105/tpc.011825 12782737 PMC156380

[B59] Benhamed M Bertrand C Servet C Zhou DX *Arabidopsis* GCN5, HD1, and TAF1/HAF2 interact to regulate histone acetylation required for light-responsive gene expression Plant Cell 2006 18 2893 2903 10.1105/tpc.106.043489 17085686 PMC1693931

[B60] Staffa A Cochrane A Identification of positive and negative splicing regulatory elements within the terminal tat-rev exon of human immunodeficiency virus type 1 Mol Cell Biol 1995 15 4597 4605 7623851 10.1128/mcb.15.8.4597 PMC230700

[B61] Yeakley JM Hedjran F Morfin JP Merillat N Rosenfeld MG Emeson RB Control of calcitonin/calcitonin gene-related peptide pre-mRNA processing by constitutive intron and exon elements Mol Cell Biol 1993 13 5999 6011 8413203 10.1128/mcb.13.10.5999-6011.1993 PMC364659

[B62] Tanaka K Watakabe A Shimura Y Polypurine sequences within a downstream exon function as a splicing enhancer Mol Cell Biol 1994 14 1347 1354 8289812 10.1128/mcb.14.2.1347-1354.1994 PMC358489

[B63] McCullough AJ Schuler MA Intronic and exonic sequences modulate 5′ splice site selection in plant nuclei Nucl Acids Res 1997 25 1071 1077 10.1093/nar/25.5.1071 9023120 PMC146543

[B64] Pertea M Mount SM Salzberg SL A computational survey of candidate exonic splicing enhancer motifs in the model plant *Arabidopsis thaliana* BMC Bioinformatics 2007 8 159 10.1186/1471-2105-8-159 17517127 PMC1892810

[B65] Arabidopsis 2010 project to characterize pre-mRNA splicing signals [http://www.life.umd.edu/labs/mount/2010-splicing/ESEs.html]

[B66] Yeakley JM Morfin JP Rosenfeld MG Fu XD A complex of nuclear proteins mediates SR protein binding to a purine-rich splicing enhancer Proc Natl Acad Sci USA 1996 93 7582 7587 10.1073/pnas.93.15.7582 8755518 PMC38789

[B67] Ramchatesingh J Zahler AM Neugebauer KM Roth MB Cooper TA A subset of SR proteins activates splicing of the cardiac troponin T alternative exon by direct interactions with an exonic enhancer Mol Cell Biol 1995 15 4898 4907 7651409 10.1128/mcb.15.9.4898 PMC230736

[B68] Thomas J Palusa SG Prasad KV Ali GS Surabhi GK Ben-Hur A Abdel-Ghany SE Reddy AS Identification of an intronic splicing regulatory element involved in auto-regulation of alternative splicing of SCL33 pre-mRNA Plant J 2012 72 935 946 10.1111/tpj.12004 22913769

[B69] Will CL Urlaub H Achsel T Gentzel M Wilm M Luhrmann R Characterization of novel SF3b and 17S U2 snRNP proteins, including a human Prp5p homologue and an SF3b DEAD-box protein EMBO J 2002 21 4978 4988 10.1093/emboj/cdf480 12234937 PMC126279

[B70] Manley JL Tacke R SR proteins and splicing control Gene Dev 1996 10 1569 1579 10.1101/gad.10.13.1569 8682289

[B71] Graveley BR Sorting out the complexity of SR protein functions RNA 2000 6 1197 1211 10.1017/S1355838200000960 10999598 PMC1369994

[B72] Imaizumi T Kadota A Hasebe M Wada M Cryptochrome light signals control development to suppress auxin sensitivity in the moss *Physcomitrella patens* Plant Cell 2002 14 373 386 10.1105/tpc.010388 11884681 PMC152919

[B73] Cosmoss database http://cosmoss.org/

[B74] Phytozome http://www.phytozome.net/physcomitrella.php

[B75] Kent WJ BLAT-The BLAST-like alignment tool Genome Res 2002 12 656 664 11932250 10.1101/gr.229202 PMC187518

[B76] Read analysis & comparison kit in Java http://rackj.sourceforge.net/

[B77] Gene ontology browsing utility http://gobu.openfoundry.org/

[B78] Lin W-D Chen Y-C Ho J-M Hsiao C-D GOBU: toward an integration interface for biological objects J Inf Sci Eng 2006 22 19 29

[B79] Chen Y-C Chen Y-C Lin W-D Hsiao C-D Chiu H-W Ho J-M Bio301: a web-based EST annotation pipeline that facilitates functional comparison studies ISRN Bioinformatics 2012 2012 139842 10.5402/2012/139842 PMC4407203 25969743

[B80] Alexa A Rahnenfuhrer J Lengauer T Improved scoring of functional groups from gene expression data by decorrelating GO graph structure Bioinformatics 2006 22 1600 1607 10.1093/bioinformatics/btl140 16606683

[B81] Saldanha AJ Java Treeview–extensible visualization of microarray data Bioinformatics 2004 20 3246 3248 10.1093/bioinformatics/bth349 15180930

[B82] National Center for Biotechnology Information-Sequence Read Archive database[http://www.ncbi.nlm.nih.gov/sra]

