# Supplementary material for: Genome-wide analysis of light-regulated alternative splicing mediated by photoreceptors in Physcomitrella patens
Source: Genome Biol. 2014 Jan 7;15(1):R10. doi: 10.1186/gb-2014-15-1-r10 (PMC4054894; doi:10.1186/gb-2014-15-1-r10)
Supplement: Additional file 1 — Contains Supplementary Figures S1 to S5 and Supplementary Tables S1 to S3. [file gb-2014-15-1-r10-S1.pdf]

Supplementary Figures and Tables

Genome-wide analysis of light-regulated alternative splicing mediated by photoreceptors in *Physcomitrella patens*

Hshin-Ping Wu, Yi-shin Su, Hsiu-Chen Chen, Yu-Rong Chen, Chia-Chen Wu, Wen-Dar Lin, and Shih-Long Tu

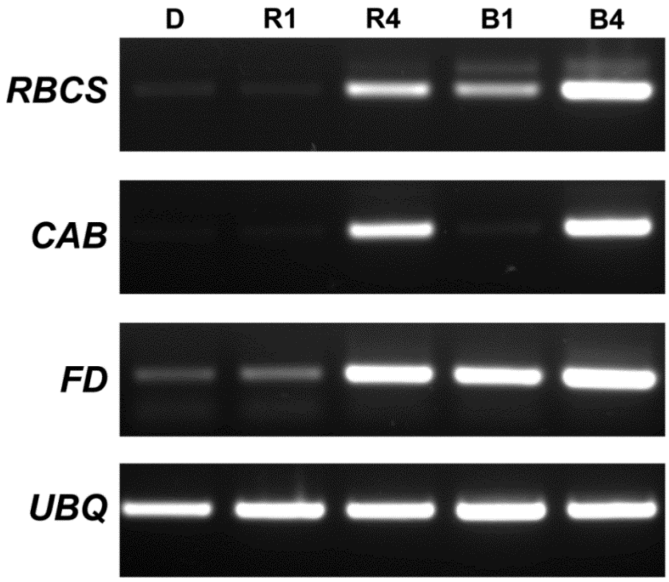

Supplementary Figure 1. Expression of light-responsive marker genes. RT-PCR analysis of pooled RNA from control (D), 1-h constant red light (Rc) (R1), 4-h Rc (R4), 1-h constant blue light (Bc) (B1) and 4-h Bc (B4) samples. Primer sets specific for *PpRBCS, PpCAB, PpFD,* and *PpUBQ* transcripts were employed (see Additional file 7). *PpUBQ* was a control.

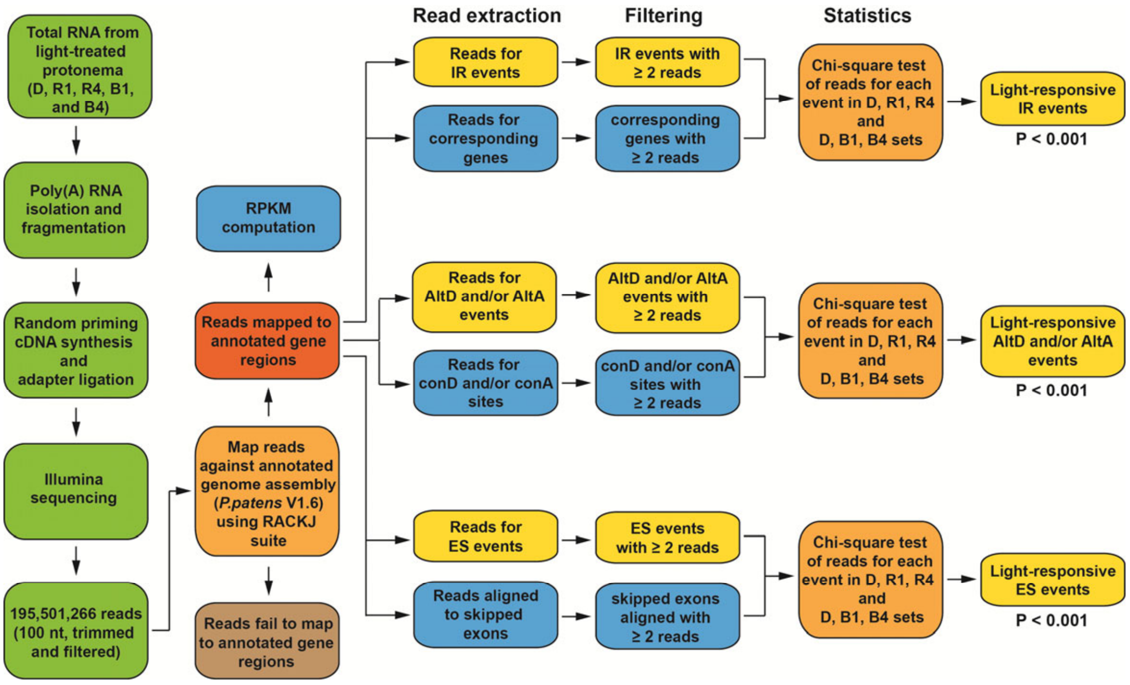

Supplementary Figure 2. Flowchart for RNA-sequencing and data analysis. Procedure for preparation of cDNA libraries and pipeline for data analysis were shown. RPKM, reads per kilobase of exon model per million mapped reads. D, Dark-grown. R1, 1-h constant red light (Rc). R4, 4-h Rc. B1, 1-h constant blue light (Bc). B4, 4-h Bc. IR, intron retention. AltD, alternative donor site. AltA, alternative acceptor site. conD, constitutive donor site. conA, constitutive acceptor site. ES, exon skipping.

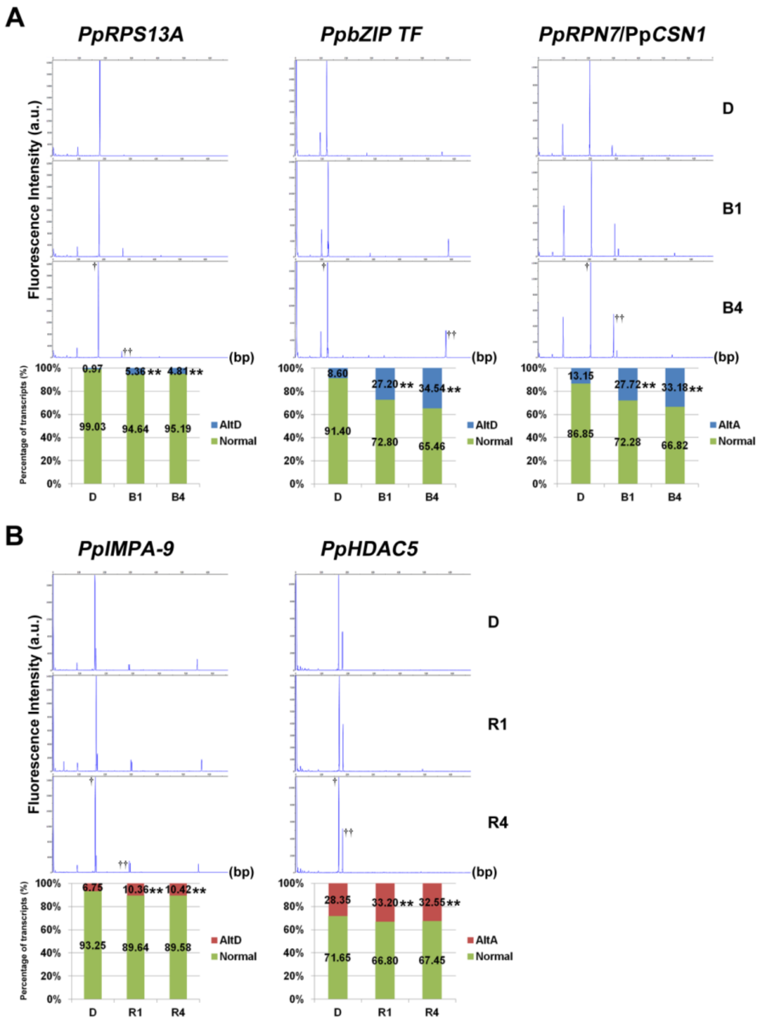

Supplementary Figure 3. Validation of light-regulated alternative donor and acceptor sites. High-resolution RT-PCR was used for determining the level of selected AltD and AltA isoforms. Pooled RNA from control (dark-grown; D), 1-h Rc-treated (R1), 4-h Rc-treated (R4), 1-h Bc-treated (B1) and 4-h Bc-treated (B4) samples were used for RT-PCR with primer sets designed for amplifying the AltD and AltA regions (see Additional data file 7). DNA fragments were separated on an ABI3730 DNA Analyzer with 3 technical repeats. Integrated peak areas of RT-PCR products identified with expected sizes were used as the relative expression level. Percentage of each AS isoform was calculated by dividing by the sum of all detected transcripts. ANOVA was used to determine the significance in differences between control, 1- and 4-h light treated samples. Significance: ** P< 0.01; * 0.01>P<0.05. Events were selected from blue (**A**) and red light (**B**) samples. †, PCR products from constitutive splicing; ††, PCR products from alternative splicing.

A

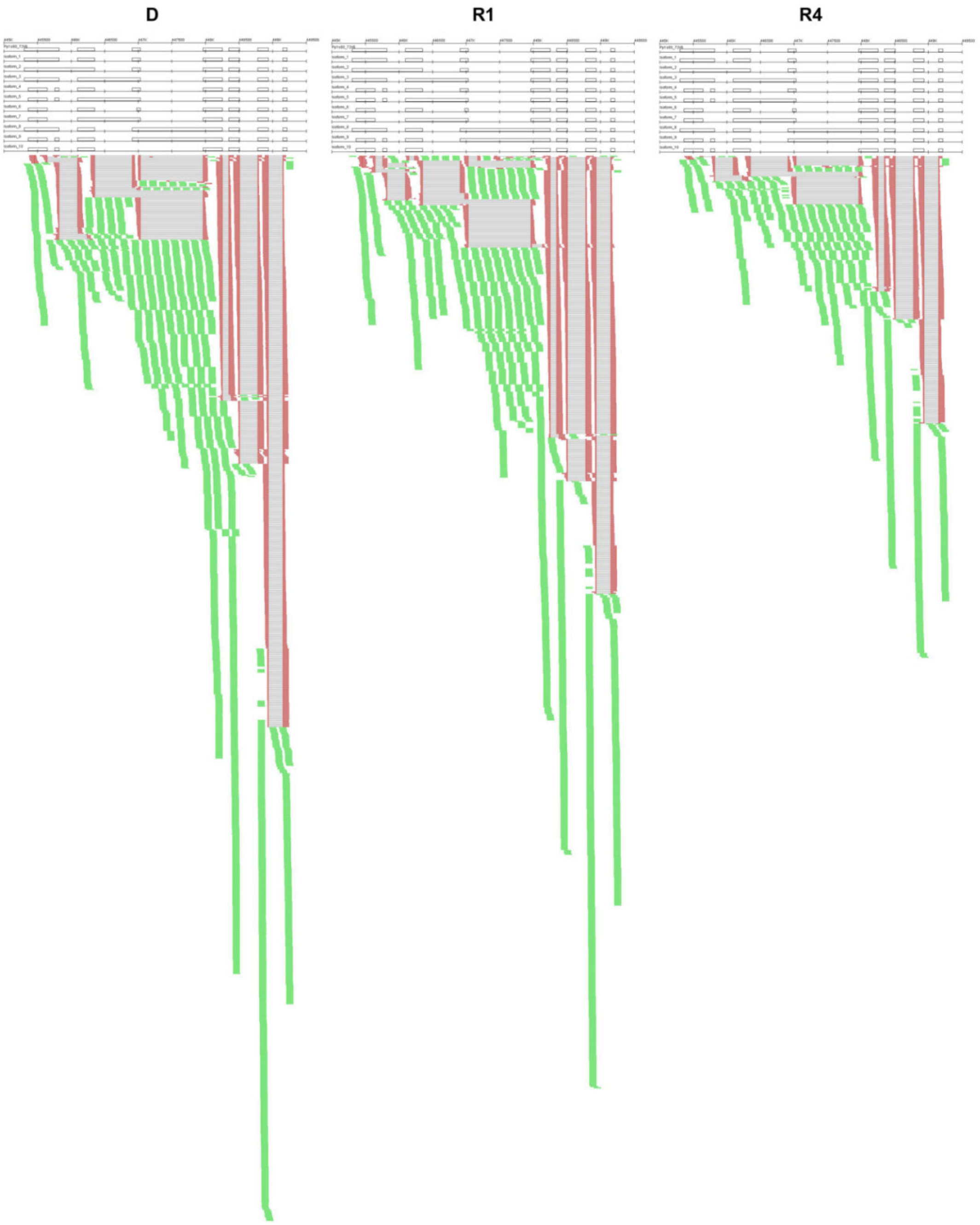

B

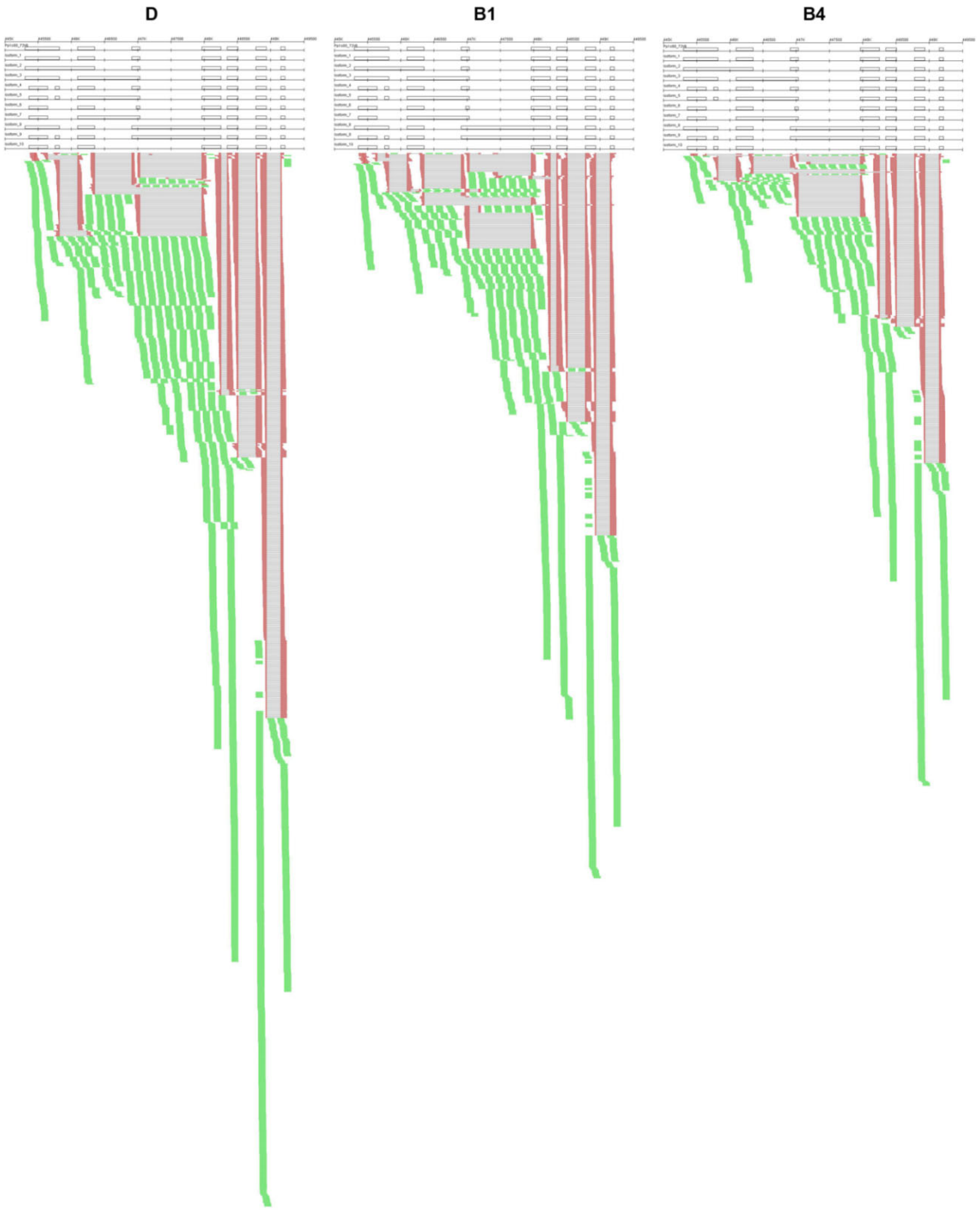

Supplementary Figure 4. Mapping results of revised *PpHYH2* from RNA-seq data of dark-grown (D), 1-h Rc-treated (R1), 4-h Rc-treated (R4), 1-h Bc-treated (B1) and 4-h Bc-treated (B4) samples (**A**) Comparison of D, R1 and R4. (**B**) Comparison of D, B1 and B4.

PpSR34A
PpSCL30A
PpSCL42
PpRPP3A

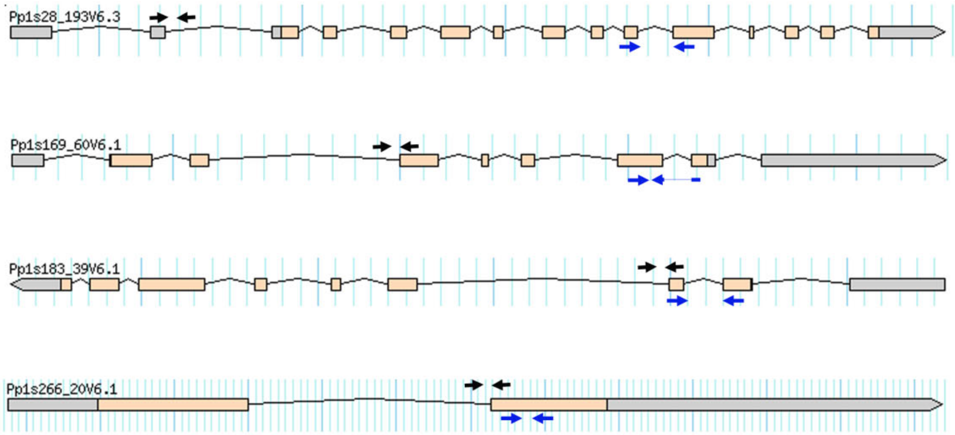

Supplementary Figure 5. Schematic diagrams of gene models and primers for detecting IR of SR protein and ribosomal protein genes. Representative transcripts of *PpSR34A*, *PpSCL30A*, *PpSCL42* and *PpRPP3A* were plotted. Primers designed for detecting IR level were indicated in black arrows. Primers used for detecting total transcript level were indicated in blue arrows.

A

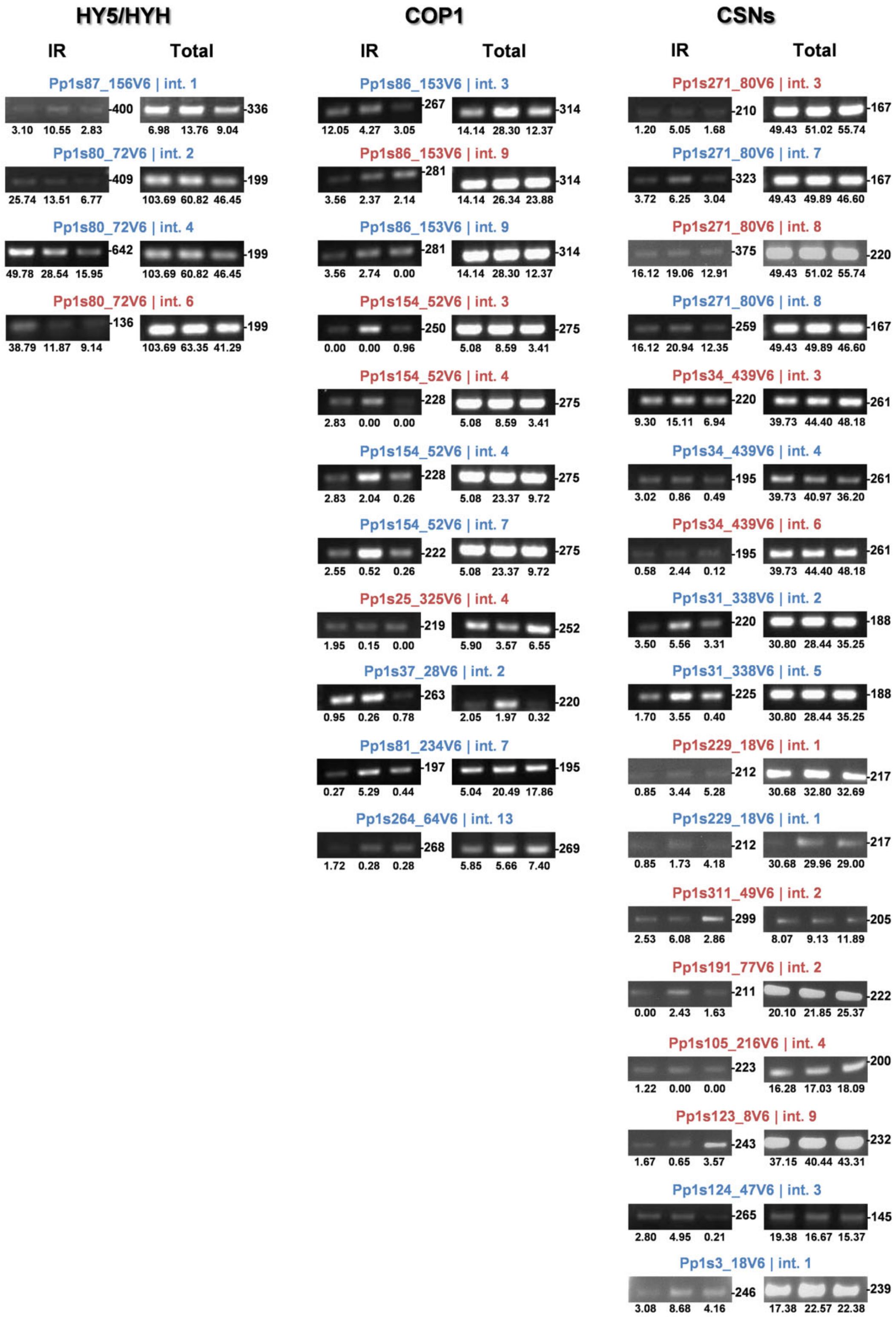
HY5/HYH
COP1
CSNs
IR
Total
Pp1s87_156V6 | int. 1
400
3.10 10.55 2.83
336
6.98 13.76 9.04
Pp1s80_72V6 | int. 2
409
25.74 13.51 6.77
199
103.69 60.82 46.45
Pp1s80_72V6 | int. 4
642
49.78 28.54 15.95
199
103.69 60.82 46.45
Pp1s80_72V6 | int. 6
136
38.79 11.87 9.14
199
103.69 63.35 41.29
Pp1s86_153V6 | int. 3
267
12.05 4.27 3.05
314
14.14 28.30 12.37
Pp1s86_153V6 | int. 9
281
3.56 2.37 2.14
314
14.14 26.34 23.88
Pp1s86_153V6 | int. 9
281
3.56 2.74 0.00
314
14.14 28.30 12.37
Pp1s154_52V6 | int. 3
250
0.00 0.00 0.96
275
5.08 8.59 3.41
Pp1s154_52V6 | int. 4
228
2.83 0.00 0.00
275
5.08 8.59 3.41
Pp1s154_52V6 | int. 4
228
2.83 2.04 0.26
275
5.08 23.37 9.72
Pp1s154_52V6 | int. 7
222
2.55 0.52 0.26
275
5.08 23.37 9.72
Pp1s25_325V6 | int. 4
219
1.95 0.15 0.00
252
5.90 3.57 6.55
Pp1s37_28V6 | int. 2
263
0.95 0.26 0.78
220
2.05 1.97 0.32
Pp1s81_234V6 | int. 7
197
0.27 5.29 0.44
195
5.04 20.49 17.86
Pp1s264_64V6 | int. 13
268
1.72 0.28 0.28
269
5.85 5.66 7.40
Pp1s271_80V6 | int. 3
210
1.20 5.05 1.68
167
49.43 51.02 55.74
Pp1s271_80V6 | int. 7
323
3.72 6.25 3.04
167
49.43 49.89 46.60
Pp1s271_80V6 | int. 8
375
16.12 19.06 12.91
220
49.43 51.02 55.74
Pp1s271_80V6 | int. 8
259
16.12 20.94 12.35
167
49.43 49.89 46.60
Pp1s34_439V6 | int. 3
220
9.30 15.11 6.94
261
39.73 44.40 48.18
Pp1s34_439V6 | int. 4
195
3.02 0.86 0.49
261
39.73 40.97 36.20
Pp1s34_439V6 | int. 6
195
0.58 2.44 0.12
261
39.73 44.40 48.18
Pp1s31_338V6 | int. 2
220
3.50 5.56 3.31
188
30.80 28.44 35.25
Pp1s31_338V6 | int. 5
225
1.70 3.55 0.40
188
30.80 28.44 35.25
Pp1s229_18V6 | int. 1
212
0.85 3.44 5.28
217
30.68 32.80 32.69
Pp1s229_18V6 | int. 1
212
0.85 1.73 4.18
217
30.68 29.96 29.00
Pp1s311_49V6 | int. 2
299
2.53 6.08 2.86
205
8.07 9.13 11.89
Pp1s191_77V6 | int. 2
211
0.00 2.43 1.63
222
20.10 21.85 25.37
Pp1s105_216V6 | int. 4
223
1.22 0.00 0.00
200
16.28 17.03 18.09
Pp1s123_8V6 | int. 9
243
1.67 0.65 3.57
232
37.15 40.44 43.31
Pp1s124_47V6 | int. 3
265
2.80 4.95 0.21
145
19.38 16.67 15.37
Pp1s3_18V6 | int. 1
246
3.08 8.68 4.16
239
17.38 22.57 22.38

B

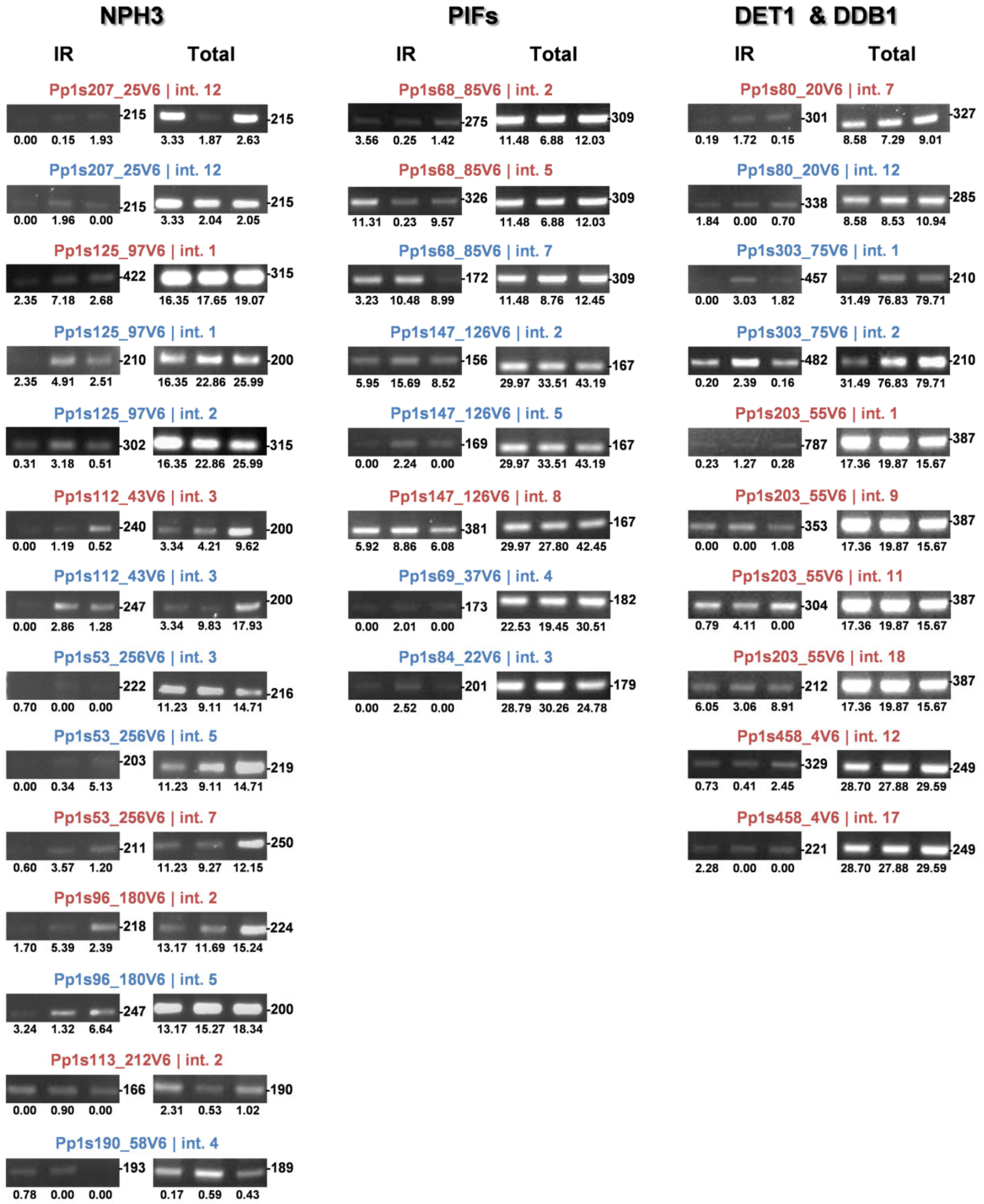

Supplementary Figure 6. RT-PCR of light signaling genes showing light-sensitive IR events. Primers designed for detecting IR level (IR, left panel) and total transcripts (Total, right panel) of each IR gene were used for RT-PCR reaction (see Additional File 7). Gene ID and the number of retained intron were shown on the top. Red and blue labels indicate that the light-sensitive IR events were detected under red and blue light, respectively. IPKM for IR level and RPKM for total transcript level were indicated in the bottom.

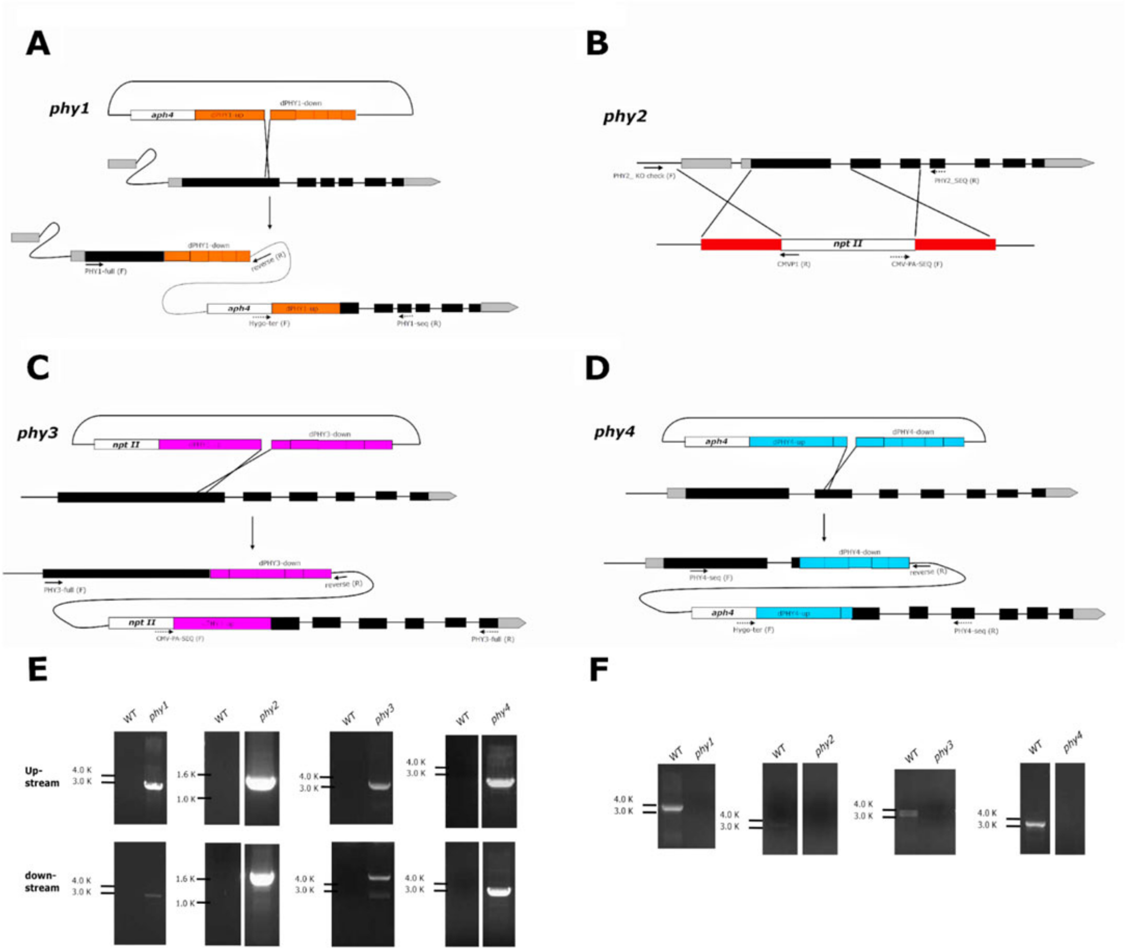

Supplementary Figure 7. Construction and verification of gene-targeted phytochrome mutant lines. Construction strategies used for generating (**A**) *phy1* (**B**) *phy2* (**C**) *phy3* (**D**) *phy4* mutants are shown. The exons of genes are shown in black. The expression cassettes of antibiotic resistance genes are located between regions homologous to the genome [orange in (**A**); red in (**B**); purple in (**C**); blue in (**D**)]. (**E**) To verify the DNA insertion in genomes of wild type (WT) and gene-disrupted mutants, primers [solid and dotted arrows in (**A**) (**B**) (**C**) (**D**)] were used to amplify upstream and downstream insertions, respectively (Additional File 7). Sizes of the upstream (up) and downstream (down) PCR products for each transgenic line were: *phy1* (2.9 kb/2.9 kb), *phy2* (1.5 kb/1.6 kb), *phy3* (3.0 kb/4.3 kb), *phy4* (2.5 kb/3.0 kb). (**F**) To verify the RNA expression of phytochrome genes in WT and gene-disrupted mutants by RT-PCR.

Supplementary Table 1. Mapping statistics of RNA-sequencing

	**D**	**R1**	**R4**	**B1**	**B4**	**Total**
Sequence reads	31,405,722	46,414,960	38,912,604	39,776,860	38,991,120	195,501,266
Mapped reads	22,588,654	32,139,792	28,209,364	27,851,810	27,606,613	138,396,233
Mapping pct. (%)[1]	71.93	69.24	72.49	70.02	70.80	70.79
Intragenic reads	19,850,289	27,919,497	24,713,906	24,101,568	24,120,706	120,705,966
Exonic reads	13,982,336	19,667,252	17,595,154	17,259,861	17,596,043	86,100,646
Intronic reads	646,168	1,078,490	882,360	879,090	776,546	4,262,654
Splice junction reads	4,411,722	5,981,274	5,218,820	4,959,277	4,818,406	25,389,499
Exon-intron reads[2]	810,063	1,192,481	1,017,572	1,003,340	929,711	4,953,167
Intergenic reads	2,738,365	4,220,295	3,495,458	3,750,242	3,485,907	17,690,267

[1] Percentage of Sequence reads mapped to the *Physcomitrella* genome
[2] Reads aligned with splice sites

Supplementary Table 2. Premature termination codon (PTC) prediction of IR events

Red light

	All	Top 1,000
IR events	70,776	1,000
IR transcripts	96,628	1,425
IR transcripts with retained intron in protein coding region	85,859	1,245
PTC in the retained intron	82,829	1,234
PTC in the downstream region	2,101	9
IR transcripts without PTC	929	2
Pct. of IR transcripts with PTC	87.89%	87.22%

Blue light

	All	Top 1,000
IR events	54,927	1,000
IR transcripts	95,245	1,452
IR transcripts with retained intron in protein coding region	84,626	1,193
PTC in the retained intron	81,638	1,177
PTC in the downstream region	2,069	10
IR transcripts without PTC	919	6
Pct. of IR transcripts with PTC	87.88%	81.75%

Supplementary Table 3. Functional enrichment of light-regulated AltD/AltA events.

Red light		Blue light	
**GO Term**	**P Value**	**GO Term**	**P Value**
CC: nucleus	4.59E-16	CC: chloroplast	1.90E-15
MF: ATP binding	2.77E-11	CC: nucleus	7.18E-15
BP: ubiquitin-dependent protein catabolic process	3.17E-09	BP: nitrogen compound metabolic process	2.91E-12
MF: protein serine/threonine kinase activity	7.29E-06	MF: ATP binding	4.33E-11
MF: magnesium ion binding	9.99E-06	MF: zinc ion binding	1.33E-09
MF: zinc ion binding	1.54E-05	MF: ligase activity	2.78E-08
CC: cytoskeleton	1.86E-05	MF: kinase activity	6.49E-08
MF: ATPase activity	5.85E-05	MF: flavin adenine dinucleotide binding	7.52E-07
MF: serine-type endopeptidase activity	6.74E-05	BP: tRNA processing	1.06E-06
BP: chromatin modification	7.44E-05	BP: ubiquitin-dependent protein catabolic process	2.73E-06
BP: cellular amino acid biosynthetic process	7.77E-05	BP: chromatin modification	4.33E-06
BP: protein phosphorylation	8.10E-05	MF: GTP binding	5.57E-06
CC: clathrin coat of trans-Golgi network vesicle	1.14E-04	MF: RNA binding	5.62E-06
BP: mRNA processing	1.36E-04	MF: magnesium ion binding	1.26E-05
BP: unfolded protein binding	1.59E-04	BP: protein modification process	1.32E-05
BP: protein transport	1.63E-04	BP: mRNA transport	6.62E-05
MF: enzyme binding	2.41E-04	MF: RNA polymerase II transcription mediator activity	6.71E-05
MF: oxidoreductase activity, acting on a sulfur group of donors, oxygen as acceptor	2.59E-04	MF: oxidoreductase activity, acting on the CH-NH2 group of donors	9.97E-05
MF: aminopeptidase activity	3.33E-04	MF: ATPase activity	1.29E-04
MF: galactosidase activity	4.48E-04	MF: solute:cation symporter activity	1.36E-04
MF: flavin adenine dinucleotide binding	5.29E-04	MF: exopeptidase activity	3.39E-04
MF: carbohydrate kinase activity	7.66E-04	CC: cytoplasmic vesicle	4.81E-04
MF: actin filament binding	8.42E-04	MF: carbon-carbon lyase activity	8.63E-04

A total of 2,678 genes and 2,679 genes from red- and blue-light samples were submitted to the GOBU functional enrichment tool, resulting in 1,644 and 1,685 unique genes with annotations. Terms are ranked by P value of overpresentation and are included in list if P < 1E-03. BP: Biological Process; MF: Molecular Function; CC: Cellular Component.
